# Genomic constraint and hypervariability correlate with fitness effects in elite tetraploid potato breeding material

**DOI:** 10.1371/journal.pgen.1012271

**Published:** 2026-08-11

**Authors:** Trine Aalborg, Zihui Ding, Yaoyao Wu, Elsa Sverrisdóttir, Kåre Lehmann Nielsen, Guillaume P. Ramstein

**Affiliations:** 1 Department of Chemistry and Bioscience, Aalborg University, Aalborg, Denmark; 2 State Key Laboratory of Crop Genetics and Germplasm Enhancement and Utilization, College of Horticulture, Nanjing Agricultural University, Nanjing, China; 3 KMC Amba, Brande, Denmark; 4 Center for Quantitative Genetics and Genomics, Aarhus University, Aarhus, Denmark; The University of Kansas, UNITED STATES OF AMERICA

## Abstract

Tetraploid potato is the most important vegetable food crop in the world. However, genetic gains from modern breeding efforts have been limited due to tetrasomic inheritance, the accumulation of deleterious mutations during domestication, and subsequent clonal propagation during modern breeding. In this study, we analyzed previously identified evolutionarily constrained genomic regions in the potato genome relative to a breeding panel of tetraploid potato clones derived from elite cultivars (the MASPOT panel). We demonstrate a significant association between derived allele frequency (DAF) and both positive (constrained) and negative (excess of substitutions relative to neutrality, i.e., hypervariable) nucleotide conservation scores (GERP scores) in the breeding panel, indicating that negative GERP scores can annotate sites affected by positive selection or other non-neutral processes affecting population allele frequency. The deep phylogeny approach can be leveraged to alleviate inbreeding in potatoes through the qualification of causative alleles in tetraploids, offering a valuable resource for potato genetic improvement by molecular breeding. Estimates of genetic load within the tetraploid clones reveal that heterozygous loads vastly exceed homozygous loads for deleterious alleles, consistent with masking of recessive mutations. Deleterious load was significantly correlated with several trait phenotypes but did not improve prediction accuracy of any traits in the tetraploid panel. This could be due to the increased complexity of tetrasomic inheritance, including more effective load complementation, and/or a higher proportion of heterozygosity in tetraploid compared to diploid potato. Intriguingly, the hypervariable load correlated significantly with several phenotypes and improved genomic prediction accuracy of yield by 5% relative to a negative control (permuted loads). This indicates that the inferred hypervariable sites from *Solanaceae* evolutionary information may contain genetic information relevant for breeding, and that, in contrast to current practice, it is relevant to include and further explore sites with negative GERP scores in general.

## Author summary

Potato is the world’s most important vegetable crop, yet breeding progress has been slow. This is largely due to the extreme genetic complexity of cultivated potato - a tetrasomic polyploid with high genetic diversity. This complex genetic architecture allows deleterious genetic variation to accumulate and persist in the modern breeding pool, complicating parent selection. We applied an evolutionary genomics framework to elite tetraploid potato cultivars to analyze genomic regions that are evolutionarily constrained and hypervariable. We found that mutation loads of evolutionary constrained and hypervariable variants correlated with some agronomic traits (e.g., dry matter content) and tested whether incorporation of this phylogenomic information could improve genomic prediction for key agronomic traits in tetraploid potato. Importantly, the hypervariable load significantly correlated with traits such as dry matter content. Moreover, incorporation of hypervariable load from *Solanaceae* evolutionary information improved prediction accuracy of yield by 5%. The study results show that evolutionary signals in the potato genome remain relevant for breeding, and both constrained and hypervariable regions provide valuable targets for understanding genetic load, improving parent selection strategies, and guiding future breeding and genome-editing efforts in potatoes.

## 1 Introduction

The tetraploid crop potato, *Solanum tuberosum* L., was domesticated from diploid wild species into landraces of varying ploidy by early agrarian societies in the Andean highlands of Southern Peru in a single domestication event approximately 8,000–10,000 years ago [1] (from here, we use landraces to describe sexually derived accessions from the original center of domestication from which founders of the European breeding pool were selected, and not all locally domesticated – including recent – clonal cultivars, which are more broadly captured under the term farmer’s cultivars). Early domestication by the indigenous peoples involved the anthropogenic selection of several traits, including tuberization. Contemporary European breeding efforts initiated following the introduction of landraces in the late 1500s A.D [2–4]. Traits under early selection included latitude-independent tuberization, a depression of cytotoxic glycoalkaloids such as solanine, and uniformity in field cultivation rather than diverse adaptation [2,3,5,6]. Following the mid-1800s European late blight epidemic, disease resistance breeding was also initiated through admixture with sympatric wild species, harboring dominant disease resistance genes [2,3]. Traditional breeding initially involved incidental selection by farmers and, later, phenotypic selection in sexual crosses. It has now advanced to extensive breeding programs, including phenotyping of elaborate field trials and genotyping of pedigreed clones for application in statistical and machine learning models for selection, i.e., genomic prediction [7]. Particularly in the tetrasomic polyploid potato, traditional breeding efforts based on phenotypic selection of parents for sexual hybridization have been poor in generating genetic gain in traits with complex genetic inheritance, such as yield [8]. Indeed, there has been almost no genetic gain in the yield of cultivars released to the commercial market in the past >100 years [9,10].

Only a limited subset of 2,622 genes (6.7%) in the potato genome were found to be under selection during early domestication [3], consistent with the Fisher-Orr geometric model, where few large-effect alleles that confer a substantial increase in trait performance are substituted in early selection [11]. Following this initial phase, improvement to produce the modern elite potato cultigen pool may follow different outcomes. For selection of novel traits or in a direction contrary to anthropogenic selection during domestication, large-effect alleles can again be targeted for substitution. For trait selection consistent with early domestication most genetic variation is however likely to be deleterious compared to the adapted reference allele of the elite potato breeding population [12,13], and phenotypic improvements for those traits will mainly be conferred by small-effect beneficial mutations [11].

However, a genetic bottleneck exists in the domestic potato crop breeding populations because of a small effective population size, as a result of limited recombination rate [5,14,15], founder effects in modern potato breeding, and the frequent use of recurrent parents in the breeding programs [16]. The European potato breeding pool genetic diversity was also greatly compromised by the loss of susceptible progenitor plant materials during the mid-1800 late blight epidemic [8]. This has resulted in the accumulation of deleterious alleles over generations, leading to symptoms of severe inbreeding depression [17]. This accumulation is also facilitated by asexual, vegetative propagation [14,18] through tubers, which allows the masking of the deleterious alleles in a heterozygous state in the polyploid genome. The accumulated genetic load (elevated proportion of deleterious alleles) and reduced capability in fixing alleles with low effective population size (and a tetrasomic polyploid genome) will, in turn, limit selection efficiency and attainable genetic gains in breeding populations, creating a genetic bottleneck [13,19,20]. Additionally, there is a non-linear relationship between nucleotide conservations and fitness effects of nucleotide variants at the small effective population sizes characteristic of modern potato breeding [21]. Hence, mutations under moderate selection are rarely substituted in the population, as decreasing effective population size is associated with increased rates of genetic drift [21] — if the allele effect on fitness is smaller than the inverse effective population size it is genetic drift rather than selection that drives allele frequency changes. This implies that purging of deleterious variants is inefficient, while the rare beneficial variants are increasingly lost to genetic drift and do not get fixed in the population [19].

The elite tetraploid potato cultivars found in the commercial market display highly disparate phenotypic profiles from the earliest cultivated tetraploid landraces and their diploid wild progenitors. This improvement in the phenotypic output of a large collection of agronomic traits has been obtained through continuous anthropogenic selection. However, for several traits, including yield, there has been a limited genetic gain in the tetraploid breeding population [8–10]. Rather, results from [22] show that in the polyploid potato genome, continuous improvements through anthropogenic selection have hitherto increased the deleterious load in the elite cultivars compared to the primitively cultivated tetraploid landraces. Crop systems can vary dramatically in their response to domestication. Strong anthropogenic selection for yield and a transition to selfing have resulted in purging the genetic load from wild species to landraces and elite cultivars in sorghum and soybean [20,23,24], contrary to potato. However, the low effective population size associated with domestication bottlenecks has instead resulted in a rapid accumulation of deleterious alleles in cultivated varieties compared to wild progenitors [19] in, e.g., maize, rice, grapes, and the clonally propagated cassava [18,24–26], similar to potato. In tetraploid potato, the functional complementation of deleterious alleles and their continued accumulation during asexual propagation have effectively hindered the purging of the deleterious load through traditional and modern breeding approaches, which have instead increased their proportion and heterozygosity [22].

The persistence of a high masked genetic load in the breeding population contributes to the reduced fitness of offspring upon parent sexual hybridization. The purging of the deleterious load by purifying selection is therefore an important breeding goal [6], both in tetraploid potato breeding and in the generation of inbred diploid lines for hybrid breeding [9,27–29]. This requires estimation of the deleterious mutational load in the potato genome and its negative effect on fitness [28]. Wu et al., (2023) used deep phylogeny based on 100 whole-genome assemblies of *Solanaceae* and sister-clade *Convolvulaceae* coupled with a GERP (Genomic Evolutionary Rate Profiling) approach [30,31] to identify evolutionarily constrained genomic regions [32]. They also estimated the impact on their inclusion in genomic prediction models for agronomic traits in a diploid hybrid potato panel. Incorporation of the deleterious burden significantly improved genomic prediction accuracy of i.a. yield and tuber count, indicating that this evolutionary approach to breeding can facilitate improved parent selection for the improvement of some agronomic traits. Results also showed that genetic loads could be used to inform selection of founders for highly homozygous inbred lines, for the elimination of the high deleterious burden required to initiate hybrid potato breeding.

In this follow-up study, we utilize the phylogenomically discovered deleterious alleles based on the derived allele, i.e., the minor allele of the 100 *Solanaceae* panel from Wu et al., (2023). We analyzed fitness effects through the derived-allele frequency (DAF) and its relationship with GERP score. We also analyzed heterozygosity, gene diversity, and the inbreeding coefficient as functions of GERP score. We then assessed the effect of incorporating a weighted deleterious load in genomic prediction and genome-wide association studies (GWAS) in a panel of tetraploid potato clones to establish whether this evolutionary approach to breeding could confer similar prediction accuracy gains for tetraploid potatoes and/or influence the QTL detection power of GWAS. We evaluated traits subjected solely to anthropogenic selection, e.g., flesh color, and traits that have been subject to both natural and anthropogenic selection pressures. i.e., fitness-related traits that are also of agronomic importance such as tuber count. The evaluated traits are also characterized by various genetic architectures and heritabilities, facilitating a detailed study of the effect of genetic load incorporation in genomics-based breeding tools. Furthermore, we assessed the sequence space annotated with negative GERP scores, i.e., an apparent excess of substitutions relative to the assumption of neutrality [30]. Positive selection is a driver that can increase divergence above neutral levels and could lead to negative GERP scores [33]. However, negative GERP scores are generally not interpreted as indications of host sequence hypervariability but rather as neutral evolution, due to confounders such as alignment uncertainty or rate variance [33]. We hence apply a novel approach, based on the hypothesis that negative GERP scores can be used to annotate regions of hypervariability, possibly underpinning genetic variation relevant for breeding (e.g., sites under diversifying or clade-specific selection). The effect of incorporating a weighted hypervariable load in genomic prediction models and GWAS was also estimated. In addition, to characterize and contrast the genetic variation at constrained and hypervariable sites, we evaluated modes of complementation by dominance or epistasis.

## 2 Results

### 2.1 Deleterious and hypervariable genetic variation in the tetraploid cultigen pool

We used a panel of 768 F_1_ clones of an incomplete diallel of 18 tetraploid elite parents (the MASPOT panel) to characterize the deleterious and hypervariable genetic variation in the tetraploid cultigen pool based on the previously published deep phylogeny of 100 *Solanaceae* genomes with GERP scoring of sites in the potato reference genome [28]. Genotyping-by-sequencing (GBS) of the MASPOT panel produced a set of 97,815 high-quality biallelic SNPs (5x-60x read depth, ≤ 50% missing rate, ≥ 1% MAF) with reliable GERP scores (alignment depth ≥ 50, neutral score ≥ 2) across the potato genome (0.04% of the full diploid variant set of 267,915,549 genomic sites with a minimum alignment depth of 15 species).

**Fig 1:**
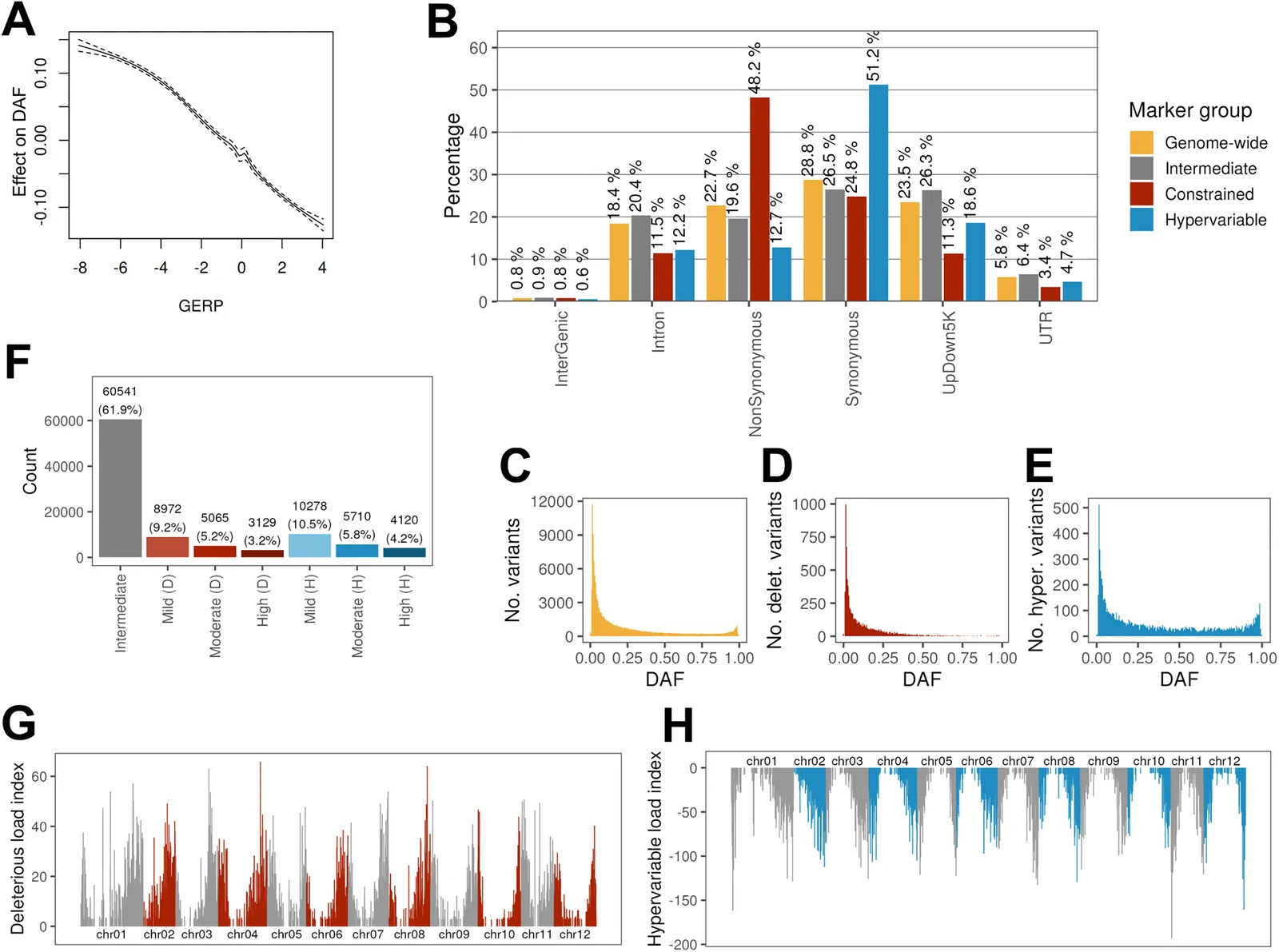
GERP scores can be used to annotate both evolutionary constraint and hypervariability. **A)** Association between the derived allele frequency (DAF) in the MASPOT panel and GERP fitted using a generalized additive model. The second axis shows the effect of GERP score on DAF. **B)** Distribution of SNP annotations across the potato genome, intermediate GERP score sites-mildly hypervariable sites (-5.5 < GERP < 2), constrained sites (GERP ≥ 2.75), and hypervariable sites (GERP ≤ -5.5). **C)** Histogram of DAF in the MASPOT panel. **D)** DAF spectrum of inferred constrained sites (GERP ≥ 2.75) in the MASPOT panel. **E)** DAF spectrum of inferred hypervariable sites (GERP ≤ -5.5) in the MASPOT panel. **F)** Distribution of SNPs according to GERP threshold (thresholds: highly deleterious [GERP ≥ 3.5], moderately deleterious [2.75 ≤ GERP < 3.5], mildly deleterious [2 ≤ GERP < 2.75], intermediate [-4 < GERP < 2], mildly hypervariable [-5.5 < GERP ≤ -4], moderately hypervariable [-7 < GERP ≤ -5.5], and highly hypervariable [GERP ≤ -7]). Number of SNPs in each group and percentage of all SNPs are shown above. **G)** Genome-wide distribution of deleterious mutation load indices, calculated by summing GERP scores of all deleterious alleles in non-overlapping 100 kb windows. **H)** Genome-wide distribution of hypervariable mutation load indices, calculated analogously to deleterious mutation load indices.

We investigated the association between the DAF and GERP score in the MASPOT panel using a generalized additive model (GAM) and found a significant association between GERP scores and DAF for both the positive GERP scores, indicating constraint, and the negative GERP scores, indicating hypervariability (Fig 1A). Similarly, the variance of genotypes at each site decreased at high GERP scores and increased at low GERP scores (reaching saturation) (S1 Fig), demonstrating that the association between DAF and GERP is not a genotyping artifact (e.g., due to paralogous site variants or copy number of heterozygous genotypes). The minor allele frequency in the landrace panel of Wu et al., (2023) was also significantly associated with GERP score across both the positive and negative range, indicating that it is a feature of potato evolutionary history (S2 Fig). The robust association between DAF and negative GERP scores suggests that low negative GERP scores may capture sites influenced by positive selection or other non-neutral processes affecting population differentiation or rapidly evolving sites. This is significant, as it suggests that the incorporation of the negative GERP scores could improve genomic prediction models, similar to previous results on the positive GERP scores [28]. Based on this analysis, we defined thresholds to annotate mild, moderate, and high mutational loads for both constrained (positive GERP scores) and hypervariable (negative GERP scores) sites. The hypervariable thresholds are equivalent to twice the numeric value of the constrained thresholds based on the distribution of the GERP scores (S3 Fig).

We assessed the prevalence of deleterious and hypervariable genetic variation in the tetraploid cultigen pool. A total of 8.4% of the sites included moderately-highly positive GERP scores (GERP ≥ 2.75), of which 48.2% are non-synonymous substitutions in CDS regions. This is a substantial enrichment (χ^2^ = 2612.5, *p* < 2.2*10^-16^; χ^2^-test) as the non-synonymous variants constitute only 22.7% of all probed SNPs (Fig 1B). The majority of the deleterious alleles with the threshold GERP ≥ 2.75 are also relatively rare considering the derived allele frequency distribution across all sites (Fig 1C-1D). The tetraploid genotypes show an overrepresentation of the inferred deleterious alleles in simplex heterozygous states, consistent with the observation of inbreeding symptoms in tetraploid cultivars due to the maintenance of deleterious mutational burden through masking by high levels of heterozygosity of the deleterious load [17].

Notably, we observed an enrichment (χ^2^ = 2105.8, *p* < 2.2*10^-16^; χ^2^-test) of synonymous substitutions in the CDS regions (51.2%) for sites with moderately-highly negative GERP scores (GERP ≤ -5.5, 10% of variants), compared to the genome-wide level (28.8%) (Fig 1B). This is not a result of enrichment of sSNPs (synonymous SNPs) in sites of intermediate-mildly negative GERP scores (-5.5 < GERP < 2), as these consisted of 26.5% sSNPs (Fig 1B). We also observed a markedly different DAF distribution among hypervariable variants (Fig 1E) compared to the distribution across all SNPs (Fig 1C), where the hypervariable sites are enriched for the derived allele across all heterozygous states and the homozygous state. This indicates that diversity in these locations is maintained in the breeding population, i.e., there is an enrichment of the derived allele at hypervariable sites. Furthermore, among the hypervariable sites, a mean (± standard deviation) proportion of 19.25 ± 2.05% of variants in the homozygous state were observed across all clones compared to 2.41 ± 0.77% for constrained sites and 10.71 ± 7.58% for all SNPs. Homozygosity of the derived allele is hence much better tolerated for the hypervariable variants than the deleterious variants, i.e., contrarily to deleterious alleles, derived alleles at hypervariable sites are not purged by homozygosity. This could suggest differential selection on the hypervariable sites compared to neutral genetic variation and compared to constrained sites as well, possibly by association of the derived allele with higher fitness. These observations support the proposition that the negative GERP scores can be used to annotate evolutionary rate using both its negative and positive range.

Based on GERP thresholds, most of the genetic variation in the breeding population (61.9%) is inferred to be within an intermediate GERP score range (-4 < GERP < 2), and similar levels of constrained and hypervariable sites are observed. As the stringency of the GERP threshold increases in either direction (mild, moderate, high), the proportion of constrained and hypervariable sites decreases (Fig 1F). The highly similar proportions of hypervariable and constrained sites might be unique to the genotyping experiment, as the landrace panel does not match this trend (S4 Fig). The distribution of the deleterious (GERP ≥ 2.75) and hypervariable (GERP ≤ -5.5) load indices across the potato genome are quite similar and follow the gene density [34], with the highest numerical loads in the apocentromeric regions (Fig 1G-1H).

### 2.2 Estimation of the genome-wide deleterious and hypervariable loads in the tetraploid clones

In this study we assessed multiple mutational loads, prioritized by 1) site based on GERP thresholds and 2) genotype. We compare loads for deleterious (GERP ≥ 2.75), hypervariable (GERP ≤ -5.5) and genome-wide variants. The genome-wide variants represent the effect of the sum of derived alleles. For each of these classes, we calculate a homozygous load, heterozygous load, and a genetic load. The homozygous and heterozygous loads were computed as the cumulative GERP scores across homozygous and heterozygous genotype states, respectively. To minimize the effect of inaccurate heterozygous allele dosage calls, we calculated the heterozygous loads assuming non-additivity. This was necessary as GBS sequencing data could not produce the > 60-80x read depth required to confidently genotype heterozygotes in autotetraploids [35], further complicated by the extensive gene copy number variation across the potato genome [36–39]. In general, we here consider a heterozygous genotype as any of the simplex (AAAB), duplex (AABB), and triplex (ABBB) heterozygous states. We define the individual genetic load as the additive mutational load in individual genomes based on the probability of transmitting a derived allele to offspring based on allele dosage. A key consequence of our load definition is that the load metrics scale with genotype state occurrence, i.e., the homozygous loads are strongly correlated with overall homozygosity (R = 0.97, *p* < 2.2*10^-16^ for the deleterious load, R = 0.99, *p* < 2.2*10^-16^ for the hypervariable load) and the heterozygous loads are correlated with overall heterozygosity (R = 0.98, *p* < 2.2*10^-16^ for the deleterious load, R = 0.99, *p* < 2.2*10^-16^ for the hypervariable load) (S5 Fig). This also produced strong correlations between the deleterious and hypervariable homozygous loads (R = 0.95, *p* < 2.2*10^-16^) and between the deleterious and hypervariable heterozygous loads (R = 0.96, *p* < 2.2*10^-16^) (S6 Fig).

**Fig 2:**
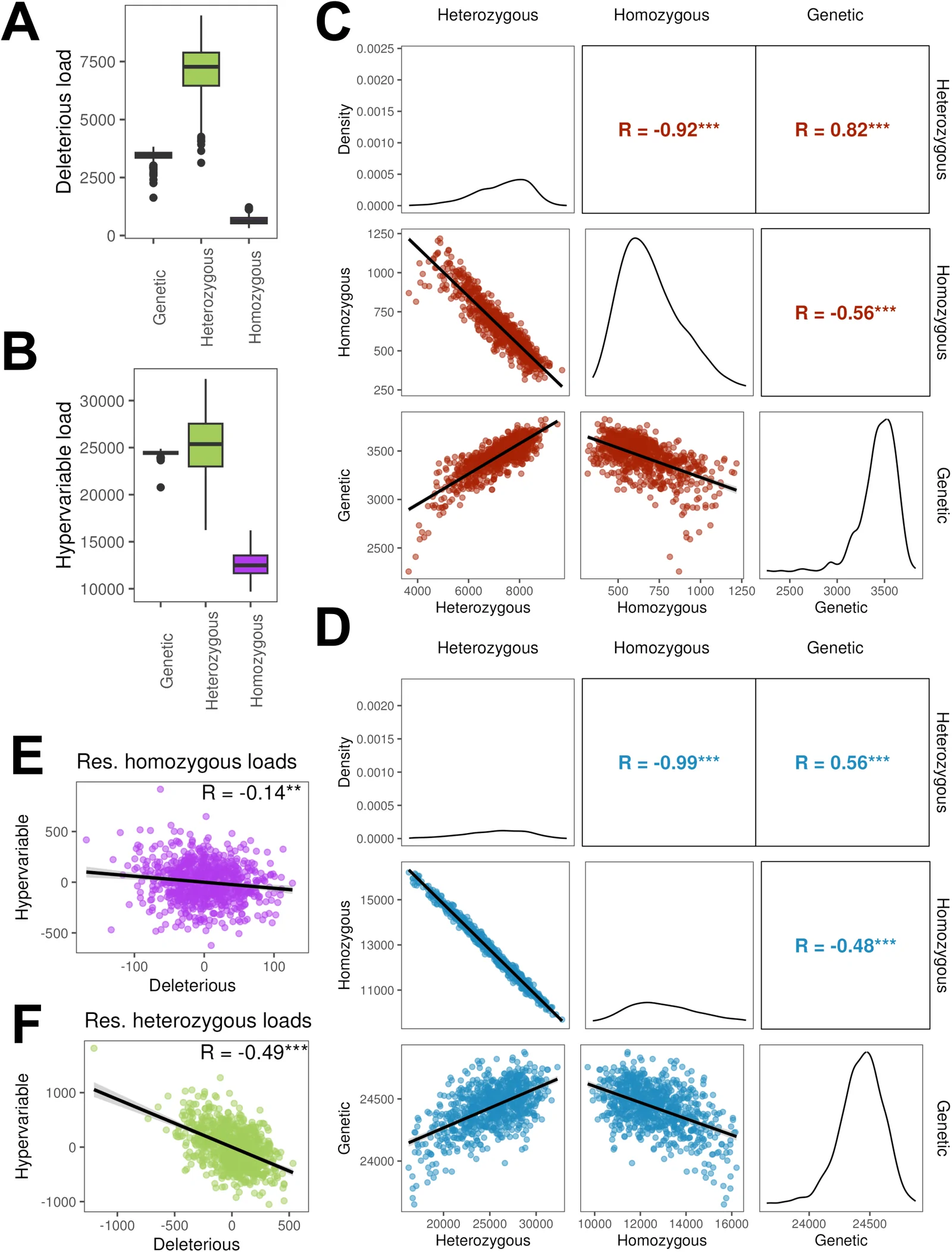
Deleterious and hypervariable mutation loads are highly correlated in tetraploid potato. **A)** Boxplot of deleterious loads across MASPOT clones, i.e., homozygous load, heterozygous load, and genetic load. **B)** Boxplot of positive hypervariable loads across MASPOT clones. **C)** Density plots of deleterious loads and correlations among genetic, heterozygous, and homozygous deleterious loads. Pearson correlation coefficients among the loads are shown. **D)** Density plots of positive hypervariable loads and correlations among hypervariable loads with Pearson correlation coefficients. **E)** Partial correlation between residual homozygous loads (deleterious and positive hypervariable) after correction for overall clone homozygosity with Pearson correlation coefficients. **F)** Partial correlation between residual heterozygous loads (deleterious and positive hypervariable) after correction for overall clone heterozygosity with Pearson correlation coefficients. **p < 0.001, ***p < 0.0001 in Pearson correlation test.

The positive heterozygous loads of both constrained and hypervariable variants are comparatively greater than the respective homozygous loads, but most pronounced for the deleterious loads. There was a ≈ 11-fold difference in heterozygous load compared to homozygous load for deleterious alleles (Fig 2A). Comparatively, there was a two-fold difference between the heterozygous and homozygous hypervariable loads (Fig 2B).

The heterozygous deleterious load is negatively correlated with the deleterious load in the homozygous state (R = -0.92, *p* < 2.2*10^-16^) (Fig 2C). The deleterious genetic load in the tetraploid panel is largely determined by the individual heterozygous load, as the homozygous load is relatively low, as observed through the strong positive correlation between the genetic load and the heterozygous load (R = 0.82, *p* < 2.2*10^-16^). There is a significant negative correlation between the deleterious genetic load and the homozygous load (R = -0.56, *p* < 2.2*10^-16^), indicating that the individuals with the lowest genetic load are also those with the lowest heterozygous load and the highest homozygous load. This is consistent with what was found in diploid potatoes [28].

For the hypervariable sites, we observe a correlation of R = -0.99 (*p* < 2.2*10^-16^) (Fig 2D) between heterozygous and homozygous loads, similar to that of the deleterious loads. However, the correlation between the hypervariable genetic load and heterozygous load is comparatively weaker than for the deleterious loads (R = 0.56, *p* < 2.2*10^-16^), suggesting that the genetic load is influenced by both the homozygous and heterozygous loads at hypervariable sites.

Consistent with the strong correlation between the deleterious and hypervariable mutational loads and the sum of derived alleles, we observed the same overall trend in correlation between homozygous, heterozygous, and genetic loads based on the sum of derived alleles (S7A Fig). For the sum of derived alleles, we observe a correlation of R = -0.99 (*p* < 2.2*10^-16^) between the heterozygous and homozygous loads, R = 0.84 (*p* < 2.2*10^-16^) between the genetic and heterozygous load, and R = -0.77 (*p* < 2.2*10^-16^) between the genetic and homozygous load. Interestingly, the correlation between genetic and homozygous load is comparatively stronger for the sum of derived alleles compared to what was found for both the deleterious and hypervariable loads, indicating that both evolutionary constraint and hypervariability influence the variation in genetic load beyond what overall homozygosity predicts. Accounting for evolutionary hypervariability similarly reduces the correlation between the genetic and heterozygous load compared to what was expected based on overall heterozygosity, while the deleterious loads largely reproduced this correlation.

We assessed the partial correlations between the deleterious and hypervariable mutational loads, i.e., between the residual homozygous loads after correcting for overall homozygosity and the residual heterozygous loads after correcting for overall heterozygosity (Fig 2E-2F). The residual deleterious and hypervariable homozygous loads are mildly, negatively correlated (R = -0.14, *p* < 0.001), while the negative correlation between the residual heterozygous loads is moderate (R = -0.49, *p* < 2.2*10^-16^). This indicates that clones with a low heterozygous deleterious load, after correcting for overall heterozygosity, generally have a high residual hypervariable heterozygous load. The reduced partial correlation between the residual homozygous loads could be due to purging deleterious alleles in the homozygous state in our population through survivor bias, as very low-fitness clones with high realized deleterious loads, and hence low plant vitality, would plausibly fail our selection criteria of 3 tubers/clone and plant viability in the field.

We identified an outlier clone (excluded from Figs 2C-2D, S7A) with a substantially lower genetic load of the derived allele compared to the remaining clones, corresponding to 15% lower than the clone with the second lowest genetic load of the derived allele (S7A–S7B Fig). This was also reproduced as a 28% lower deleterious genetic load and 12% lower positive genetic hypervariable compared to the clone with the second lowest of those respective genetic loads (S8–S9 Figs). Notably, this clone, F1-12-376-02, was lost following the first year of initial phenotyping, as it failed to satisfy propagation criteria or succumbed to disease.

### 2.3 Gene diversity of the deleterious and hypervariable loads in a tetraploid breeding population

**Fig 3:**
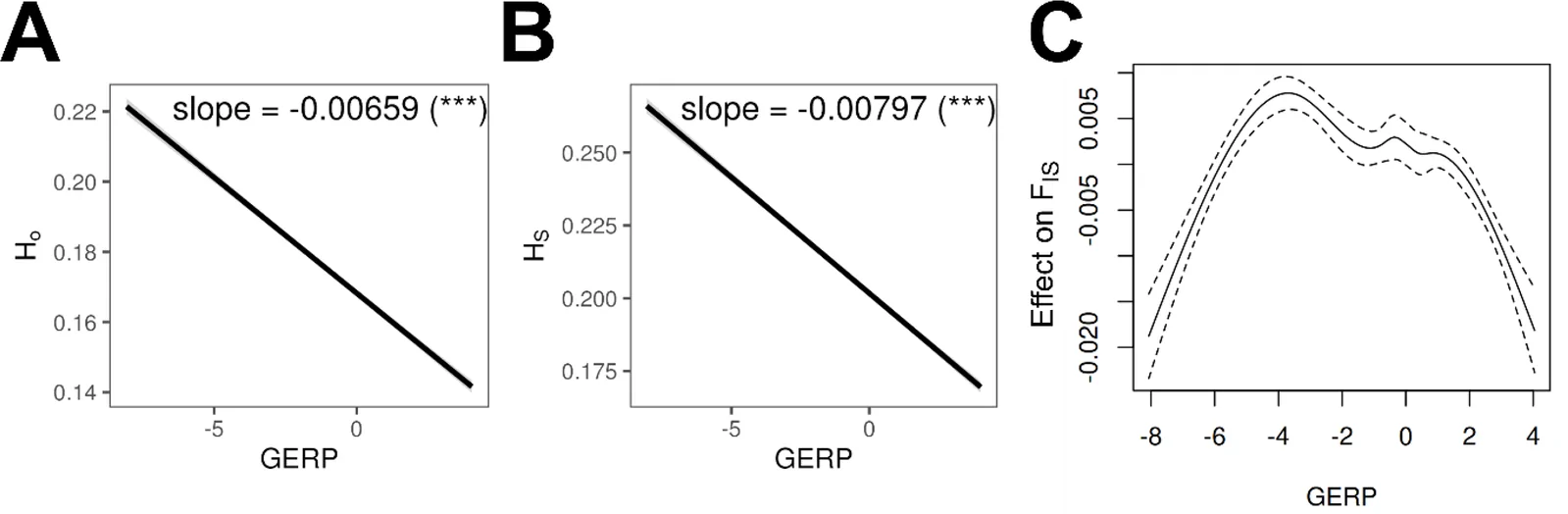
Both evolutionary constraint and hypervariability are associated with higher-than-expected heterozygosity. **A)** Linear regression of observed heterozygosity (H_O_) in the MASPOT panel as a function of GERP score. **B)** Linear regression of gene diversity (expected heterozygosity **[H_S_]**) in the MASPOT panel as a function of GERP score. **C)** Generalized additive model of inbreeding coefficient (F_IS_) in the MASPOT panel as a function of GERP score. The second axis shows the effect of GERP score on F_IS_.

To quantify the relationship between heterozygosity and GERP score, we regressed the observed heterozygosity (H_O_) and gene diversity (expected heterozygosity, H_S_) as functions of GERP score for the MASPOT panel (Fig 3A-3B). In the MASPOT diallel, H_O_ ≠ H_S_ as we imposed minimal selection criteria on the F_1_ clones (plant viability, a minimum of 3 tubers/plant), which likely removed clones with high realized deleterious genetic loads. The full MASPOT population consisted of 5,013 F_1_ clones that passed selection criteria and survived field propagation for phenotyping trials. The 768 clones considered here is a representative genotyped subsample of the selected clones [40]. Based on GAMs, linear relationships were good approximations (S10 Fig). We observed clear evidence for the severe inbreeding depression experienced by the tetraploid breeding pool [17], as H_O_ < H_S_, consistently across all variant types (S11 Fig). Both H_O_ and H_S_ decrease with increasing GERP score, consistent with a stronger purifying selection (heterozygote deficit) acting on constrained sites compared to putatively neutral sites [-4 < GERP < 2] and stronger yet compared to the hypervariable sites. We also found a similar negative linear association between GERP and H_O_ in the landrace panel of Wu et al., (2023) (S12 Fig), demonstrating that this trend is observed both in the modern breeding pool and across initial domestication. Based on H_O_ and H_S_, we computed the inbreeding coefficient (F_IS_). It had a mean value of 0.166 ± 0.149 (s.d.), i.e., demonstrating the expected inbreeding depression, but with high variability across variants (S13 Fig). Based on a GAM, the effect on F_IS_ by GERP score did not follow a linear trend. Rather, F_IS_ is lower for both constrained and hypervariable sites, compared to sites with intermediate GERP score (Fig 3C). This translates to a higher-than-expected heterozygosity of both the deleterious and hypervariable variants. For the deleterious alleles, this is consistent with what was found in diploid potatoes [28]. As both deleterious and hypervariable genetic variation is enriched for heterozygosity, we sought to evaluate how these loads are maintained through complementation to elucidate possible purging strategies for the deleterious load.

### 2.4 Complementation of deleterious and hypervariable variants in tetraploids

The percentage of heterozygous genotypes across all variants for each clone is substantial, ranging from 12% to over 49%, consistent with the extremely high heterozygosity of tetraploid potatoes [34]. We observed a significantly higher percentage of variants with a heterozygous genotype dosage (ABBB, AABB, or AAAB) for the hypervariable variants (25–49% range) compared to the putatively neutral variants [-4 < GERP < 2] (19–41% range) (t = 234.28, *p* < 2.2*10^-16^; paired Student’s t-test), and a further deficit of heterozygous genotype states for the deleterious variants (12–35% range) (t = 127.91, *p* < 2.2*10^-16^; paired Student’s t-test), consistent with H_O_ results.

**Fig 4:**
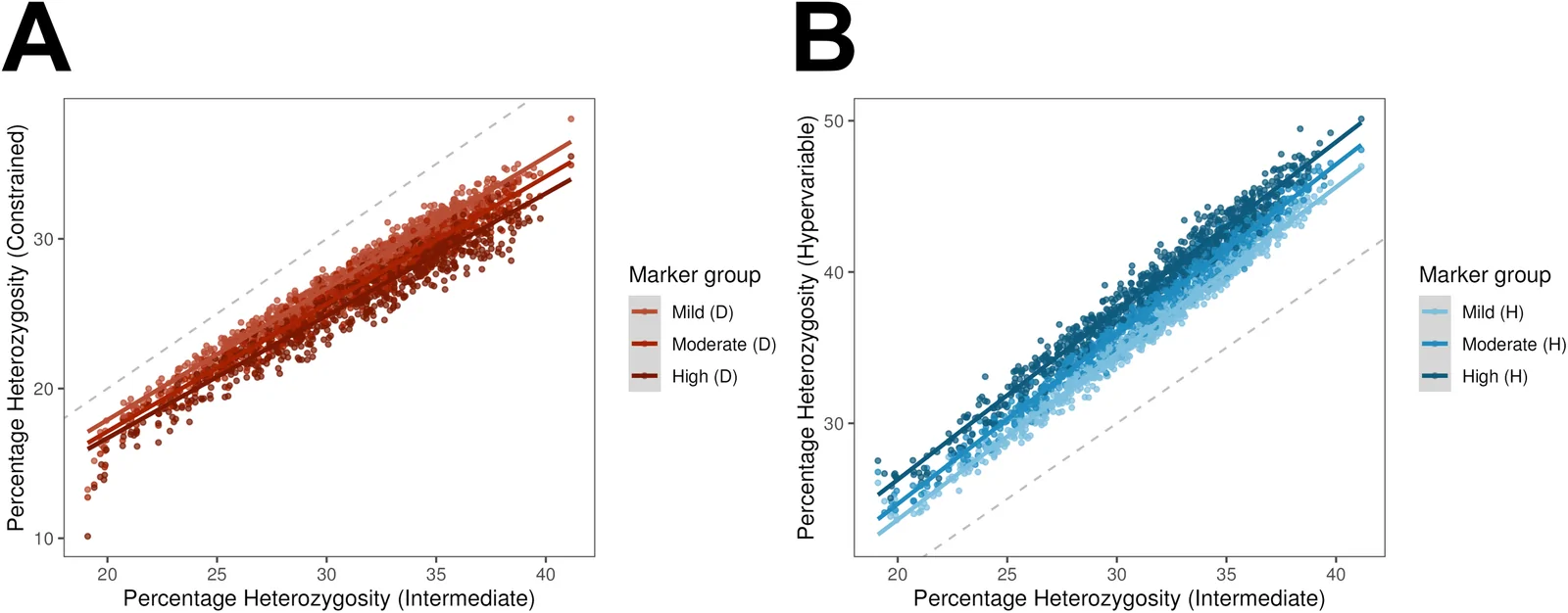
Deleterious alleles and alleles at hypervariable sites show opposing complementation patterns. **A)** Comparing the proportion of heterozygotes (simplex-triplex) of mutations of [-4 < GERP < 2] with that of mildly [2 ≤ GERP < 2.75], moderately [2.75 ≤ GERP < 3.5], and highly [GERP ≥ 3.5] deleterious mutations across the clones in the MASPOT panel. **B)** The same comparison for mildly [-5.5 < GERP ≤ -4], moderately [-7 < GERP ≤ -5.5], and highly [GERP ≤ -7] hypervariable variants. The dashed gray line indicates “*y = x*”. The solid lines represent linear regressions within groups of points and are shown with 95% confidence intervals in gray. Within each color group each point is one MASPOT F_1_ clone.

We sought to evaluate the complementation of the heterozygous inferred deleterious alleles and derived alleles at hypervariable sites in the MASPOT panel by evaluating heterozygous genotype levels relative to putatively neutral variants [41]. Under the assumption that deleterious alleles are masked by heterozygosity (i.e., simple dominance), we would expect heterozygous genotypes to occur more frequently at sites encoding deleterious alleles compared to sites with putatively neutral mutations. Interestingly, we observe that putatively neutral variants are slightly more likely in heterozygous genotypes compared to the inferred deleterious mutations across all three severity groups (mild: t = 119.04, *p*-value < 2.2*10^-16^; moderate: t = 126.29, *p*-value < 2.2*10^-16^; high: t = 112.13, *p*-value < 2.2*10^-16^; paired Student’s t-test) (Fig 4A), opposite to the expected pattern for complementation by simple dominance. The exclusion of only deleterious variants from the pool of heterozygous pool (i.e., GERP < 2) did not alter this trend (S14 Fig). Evaluation of unimputed genotypes with read depth > 20x also reproduced the same overall tendency, indicating that it is not an artifact of low accuracy genotyping (S15A Fig).

In contrast, the hypervariable variants are more likely to be found in a heterozygous state compared to putatively neutral variants, and the frequency of heterozygous genotypes increases with severity group (mild: t = -194.03, *p*-value < 2.2*10^-16^; moderate: t = -191.58, *p*-value < 2.2*10^-16^; high: t = -225.76, *p*-value < 2.2*10^-16^; paired Student’s t-test) (Fig 4B). This was also reproduced for high-read depth genotypes only (S15B Fig).

We also tested how the ratio of homozygosity changed with GERP scores across both constrained and hypervariable sites (S16A–S16B Fig). The ratio of homozygosity, like heterozygosity, depreciated significantly with severity group for constrained sites and increased significantly with severity group for hypervariable sites.

In concert, these results indicate that in the MASPOT panel, we do not observe evidence of simple dominance being the main mode of complementation of the deleterious load. However, for the hypervariable sites, there is an enrichment in heterozygosity of the derived allele, consistent with the observed DAF distribution (Fig 1G).

### 2.5 Weighted deleterious and hypervariable loads in genomic prediction

**Fig 5:**
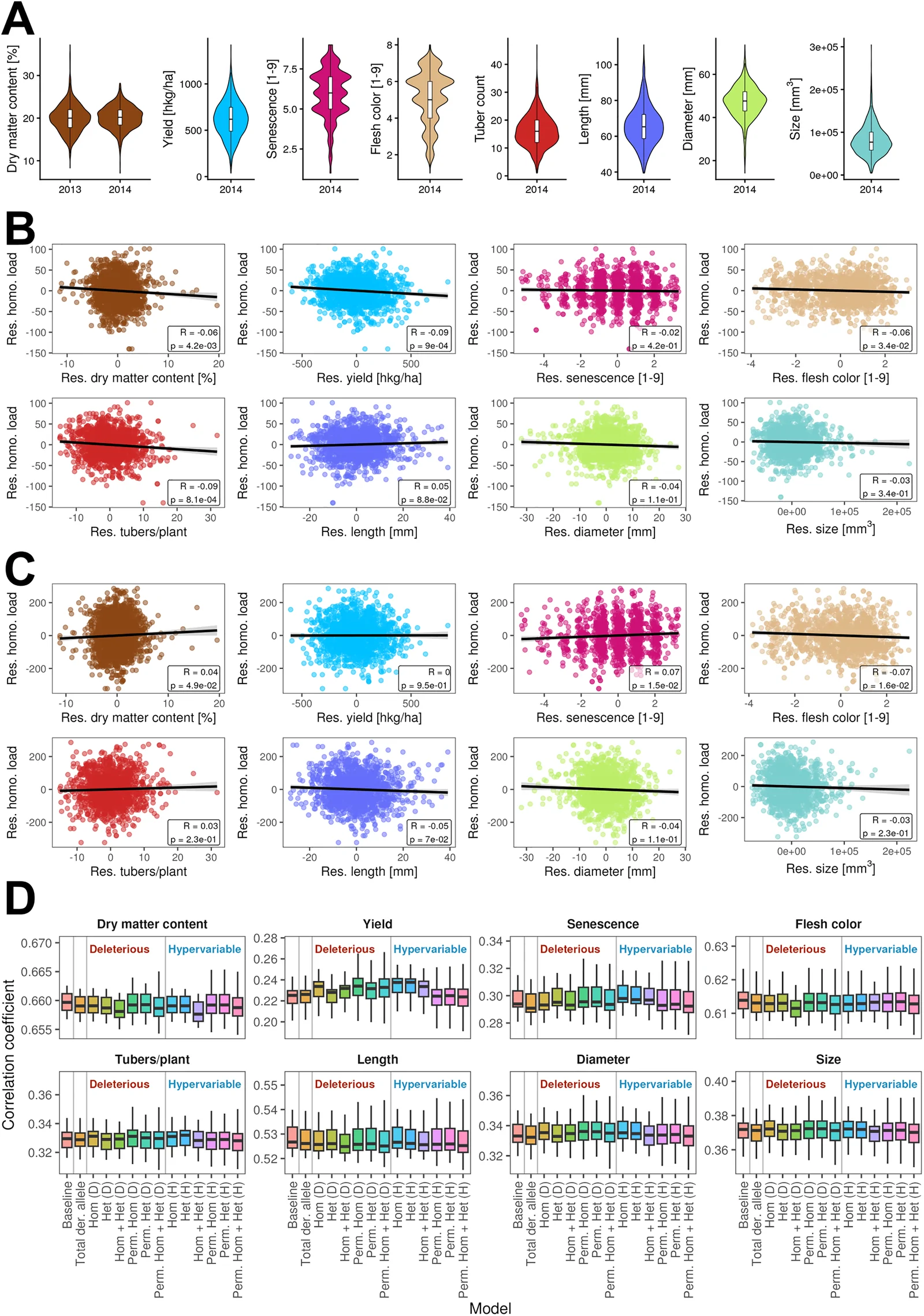
Incorporation of mutational loads in genomic prediction has trait- and load-dependent effects on prediction accuracy in tetraploid potato. **A)** Phenotypic distribution of dry matter content, yield, senescence, flesh color, tuber count, tuber length, diameter, and size in the MASPOT panel. **B)** Partial correlations between clone phenotype and the per-individual residual homozygous deleterious load (after fitting the heterozygous load and correcting for population structure [fitting principal components 1-3 of the genomic relationship matrix]) among the MASPOT panel clones for each trait. Pearson partial correlation coefficients and Pearson partial correlation test *p*-values are shown (calculated with the *pcor.test()* function of the ppcor package (v1.1) [42]). The solid black line indicates the linear regression between phenotype and residual load. Gray shadows indicate 95% confidence intervals. **C)** Partial correlations between clone phenotype and the per-individual residual positive homozygous hypervariable load (after fitting the heterozygous load and correcting for population structure [fitting principal components 1-3 of the genomic relationship matrix]) among the MASPOT panel clones for each trait. Pearson partial correlation coefficients and Pearson partial correlation test *p*-values are shown. The solid black line indicates the linear regression between phenotype and residual load. Gray shadows indicate 95% confidence intervals. **D)** Genomic prediction correlation coefficient of the eight traits using the baseline model (without fitting any loads), the total derived alleles model (fitting the number of derived alleles), the deleterious load models (fitting the homozygous and/or heterozygous deleterious loads), the permuted deleterious load models (fitting the randomly permuted scores), the hypervariable load models (fitting the homozygous and/or heterozygous hypervariable loads), and the permuted hypervariable load models (fitting the randomly permuted scores).

To evaluate the impact of incorporating weighted deleterious and hypervariable loads in genomic prediction models, we analyzed a total of eight agronomic traits: Dry matter content (h^2^ = 0.35), yield (h^2^ = 0.193), senescence (h^2^ = 0.282), flesh color (h^2^ = 0.455), tuber count (h^2^ = 0.267), tuber length (h^2^ = 0.482), diameter (h^2^ = 0.344), and size (h^2^ = 0.279) (Fig 5A).

Several trait phenotypes were significantly correlated with the residual homozygous deleterious load (after fitting the heterozygous load, to account for their correlation) (Figs 5B and S17). These traits include tuber dry matter content (R = -0.06), yield (R = -0.09, flesh color (R = -0.06), and tuber count (R = -0.09). This indicates that for these traits, the deleterious mutational load is significantly associated with trait phenotypic output and could impact tetraploid potato breeding models. For the hypervariable homozygous load and trait phenotypes we observed significant partial correlations for only dry matter content (R = 0.04) and flesh color (R = -0.07, respectively) and a correlation between the residual heterozygous hypervariable load and flesh color (R = -0.08) (Figs 5C and S18), likely due to the extreme correlation between the heterozygous and homozygous hypervariable loads (S6 Fig). The total count of derived alleles per clone correlated with dry matter content (R = -0.15), flesh color (R = -0.12), and tuber count (R = -0.08) (S19 Fig), indicating that the mean effect of a derived allele genome-wide might possibly explain part of the observed correlations between deleterious homozygous load and these traits.

We evaluated the impact of both the deleterious and hypervariable mutation loads on genomic prediction of the collection of agronomic traits (Fig 5D). Performance was evaluated relative to a baseline model that accounts for additive genomic relationships without including effects of either deleterious or hypervariable mutations and a model incorporating the sum of derived alleles for each clone, accounting for the mean effect of the derived allele. The obtained mean prediction correlation coefficients using leave-nine-families-out (LNFO) cross-validation for the baseline models were 0.66, 0.22, 0.30, 0.61, 0.33, 0.53, 0.33, and 0.37 for dry matter content, yield, senescence, flesh color, tubers/plant, length, diameter, and size, respectively. Significant covariate coefficients could only be inferred for the deleterious homozygous load and both the homozygous and heterozygous hypervariable loads for yield (Fig 5D, S1–S8 Tables). Unexpectedly, based on the partial correlations between loads and phenotypes, the incorporation of the homozygous deleterious load increased the prediction accuracy compared to the baseline model, but did not translate into an improved yield prediction accuracy compared to the permuted load, while both hypervariable loads improved yield prediction accuracy by 4.8% for the homozygous load (corrected *p*-value = 0.042) and 4.6% for the heterozygous load (n.s.) (Fig 5D). There was not a similar increase in prediction accuracy compared to the baseline model using a model when incorporating the sum of derived alleles, indicating that the observed improvement is not merely due to the inclusion of the accumulated mean effect of the derived allele genome-wide, but rather an effect of the loads. Interestingly, we see disparate effects of the hypervariable and deleterious loads on prediction accuracy. This indicates that they capture different signals – as was also expected by their differences in measures of heterozygosity and DAFs.

We also evaluated the impact of incorporating weighted deleterious and hypervariable loads as covariates in genome-wide association studies (GWAS) of the tetraploid cultivars for the same eight traits (S20–S27 Figs), compared to a baseline model without mutational loads and one incorporating the sum of derived alleles. We identified trait QTLs corresponding to previous MASPOT GWAS analyses of the same traits [43] and two known QTLs for traits flesh color and tuber diameter, previously unassessed for the MASPOT panel: the major *Y* locus on chromosome 3 (42.88 Mb), controlling the accumulation of carotenoid photoreceptive pigments in the tubers for flesh color [44–47], and the *OFP20* gene of the chromosome 10 *R*_*0*_ locus [48,49] influencing both tuber length and diameter, respectively. A list of significant SNP associations for the baseline model can be found in S1 Material, and the MAF and SNP heritability of the most significant variant of each QTL in S2 Material. No significant trait QTLs were found for yield, tubers/plant, or tuber size. Based on QQ-plots of -log_10_(*p*-value) of the baseline model compared to the various load-incorporating models (S28–S35 Figs), we only observed an increase in association power (reduction of lowest *p*-values) for yield – however, not sufficiently to generate significant trait associations for this complex, low heritability trait. These results are consistent with those observed for genomic prediction, where only yield benefitted from incorporation of mutational loads.

## 3 Discussion

### 3.1 Negative GERP scores and hypervariability

Limited prior studies have assessed hypotheses of selection mechanisms based on negative GERP scores, due to confounders such as misalignments of the MSA (multiple sequence alignment) that can inflate substitutions relative to the neutral rate (average across genomes and genomic sites across a pre-defined neutral tree [30]). However, our significant association between DAF and GERP at both negative and positive scores, found in both the diallel MASPOT panel and the diverse landrace panel of Wu et al., (2023), indicates that the negative GERP scores carry relevant information about non-neutral evolutionary processes during potato evolutionary history, from early domestication to modern breeding. Similar to our results, Cooper et al., (2010) [50] also observed that GERP scores inversely correlate with mean DAF for positive and negative GERP scores across primate exomes. However, in diploid maize only the positive GERP range was significantly associated with MAF [51]. Assuming that the negative GERP range solely annotates neutrality and misalignments, we would not expect to identify a significant negative correlation between DAF and GERP in this range, or an enrichment in annotation types (sSNPs) and heterozygosity at low GERP thresholds compared to the intermediate GERP score range, as confounders such as misalignments would be unlikely to produce statistically significant associations. Hence, we propose that conservative negative GERP thresholds, following variant filtration based on alignment depth and neutral scores, can be used to annotate genomic hypervariability, analogous to the annotation of evolutionary constraint using positive GERP thresholds.

### 3.2 Deleterious load complementation in tetraploid potato

Masking of the deleterious burden in the heterozygous state can occur through several non-mutually exclusive mechanisms of complementation [52,53]. Our approach evaluates complementation of deleterious alleles through the accumulation of heterozygotes as a result of masking by within-locus dominant alleles, i.e., simple dominance. While we found no comparative increase in heterozygotes at constrained sites vs. sites with intermediate GERP scores – and observed instead a comparative decrease – this was found to be the case in diploid maize hybrids [41]. These results suggest that while dominance is one of the main mechanisms of complementation in diploid maize hybrids, complementation of deleterious alleles may mainly achieved by alternative modes of complementation in tetrasomic polyploid potato, which would effectively render the masked deleterious mutations recalcitrant to elimination through recombination. In maize, breeding is based on heterosis in hybrids of inbred lines [54]. Hybrid selection from different heterotic groups, with group-specific haplotypes, allows the complementation of fixed alleles through dominance [55–57] with varying degrees of complementarity across heterotic groups [58]. However, in tetrasomic polyploid potatoes, the fixation of alleles in an inbred line is largely impossible, which is also why tetraploid potato breeding cannot utilize hybrid crosses [5]. Instead, recent results of a phased pangenome for hybrid diploid potato found that deleterious mutations accumulate in clusters in the coupling phase of the potato genome and that a functional repulsion phase allele functionally complements deleterious alleles, thereby facilitating their retention in the cultigen pool [29]. This suggests an alternative mechanism of complementation of the deleterious load in tetraploid potato other than dominance only, e.g., through repulsion phase complementation [28,54,59]. Repulsion-phase complementation links beneficial and deleterious alleles within haplotypes, causing Hill-Robertson interference and inhibiting optimal combinations of alleles by recombination [60]. This reduces selection efficiency at any one locus, allowing mildly deleterious, recessive alleles to accumulate [7]. Repulsion linkage complementation can produce pseudo-overdominance and has also been observed for some QTLs in diploid rice and sorghum [55,59].

Compared to diploid potatoes, the tetraploid genome also supports a higher level of functional complementation through heterozygosity by providing additional functional gene copies for each deleterious allele [22,61]. During polyploidization and crop improvement, deleterious mutations accumulated rapidly in tetraploid potato (> 50% more deleterious mutations in tetraploid vs. diploid landraces and > 15% more in elite cultivars compared to tetraploid landraces) [22], and the deleterious alleles were greatly enriched in heterozygous states in tetraploid potatoes compared to diploids [22,61]. Based on our results, we see no evidence for such increase in heterozygotes of deleterious alleles compared to alleles at intermediate GERP score sites across the tetraploid MASPOT offspring. The proportion of heterozygotes did not increase at constrained vs. intermediate GERP score sites, so deleterious variants are unlikely to accumulate through dominance complementation. Further qualification of the basis of genetic complementation in tetraploid potato could contribute additional insights into viable strategies for purging the masked deleterious load [18] such as targeted haplotype rescue by genome editing (cisgenesis or base editing) or full functional replacement by ectopic cisgenic introduction of functional alleles. Alternatively, assuming repulsion phase complementation, using a CRISPR/Cas approach to create targeted double-stranded breaks could increase recombination rates at targeted sites, effectively breaking repulsion blocks and allowing for purging through classical breeding.

### 3.3 Concordance between diploid and tetraploid deleterious load analyses

Incorporation of deleterious genetic load in genomic prediction previously showed substantial potential for increasing genetic gain, based on results in a diploid hybrid potato panel, where prediction accuracy for fitness-related traits such as yield, plant height, and tubers per plant improved by 7.2–24.7% [28]. This has also been shown for fitness-related traits in other crops, e.g., of root yield in cassava [62], grain yield and testing weight of maize [51], and biomass and plant height in sorghum [63]. Overall, the contribution of the deleterious load to phenotypic variation, and hence to genomic prediction ability, is largely trait-dependent [51,63], and improvements to genomic prediction are generally modest (4–6%).

Inclusion of the hypervariable loads translated into a substantial improvement genomic prediction accuracy relative to the permuted loads – but only for yield (5%). This somewhat aligns with previous results obtained for the MASPOT panel, where filtering variants by functional class significantly improved prediction performance exclusively for yield, with non-synonymous variants increasing prediction performance by 12% relative to a genome-wide variant set [43]. Despite observing significant partial correlations between residual mutational loads and several trait phenotypes, incorporating evolutionary constraints in the tetraploid MASPOT panel in genomic prediction models did not result in significant improvements in prediction accuracy relative to negative controls (permuted loads). The missing translation of significant load correlations with phenotype to an effect on genomic prediction accuracy by incorporating the deleterious loads might be due to the deleterious genetic load being largely governed by the extremely high heterozygous load, as we observed an 11-fold higher deleterious heterozygous load than homozygous load, compared to a 2-fold difference for the hypervariable loads. Furthermore, it is much more complex to accurately identify the heterozygous load in a tetraploid crop, which is aggravated by low read depth GBS genotypic data. Hence, it is possible that the higher masked deleterious load in tetraploid potato compared to diploid [22,61] is limiting the load effect on genomic prediction. If this is the case, sampling of the low-fitness F_1_ clones removed from the MASPOT population, expected to have a higher realized deleterious load, could have increased the statistical power of our tests and revealed an effect of the deleterious load on genomic prediction accuracy in tetraploid potato.

Of the assessed traits, only yield and tuber count and size are expected to be strongly related to plant fitness and hence have significant correlations between phenotypic performance and homozygous deleterious load. This has also been found for fitness-related traits in other crops, e.g., for root yield in cassava [62], grain yield and ear height in maize [51], plant height in sorghum [63], and yield and plant height in barley [64], as well as for plant height, tuber size, flowering time, yield (R = -0.33), and tuber count (R = -0.22) in diploid potatoes [28]. In the tetraploid potato panel, the only traits for which the phenotypic correlation with homozygous deleterious load is significant and stronger than with total derived allele count are yield (R = -0.09) and tuber count (R = -0.09), following the expectation for phenology traits. We likely did not observe a correlation for tuber size, like in diploids, due to disparate phenotyping approaches. Tuber size was evaluated as tuber volume in this study, which was not reported in the diploid study. Instead, they analyzed tuber weight [28]. The reduced strength of correlations in the tetraploids compared to diploids could suggest that the impact of deleterious load on phenotypic performance may be modulated by ploidy level and genetic architecture in the study sample, e.g., allele frequencies, complementation effects, allele dosage effects, and genome complexity. However, the lower accuracy imputed GBS genotypes have likely also contributed to a weaker signal.

We also assessed the potential of mutational loads for parent selection for the generation of inbred lines for hybrid breeding. Consistent with previous findings in diploids, we observe a negative correlation (R = -0.56) between deleterious genetic load and the homozygous load. This suggests that clones with the lowest genetic load are also those with the highest homozygous load and lowest heterozygous load, consistent with what was observed in diploid potato [28]. As previously proposed for diploids, this implies that clones with high homozygous deleterious loads, despite possibly showing less vigorous growth, will constitute superior starting material to generate the highly homozygous inbred lines needed to initiate hybrid potato breeding [28]. We observed a stronger negative correlation between the derived allele genetic load and homozygous load (R = -0.77). While this difference might be somewhat related to the deleterious genetic load being largely controlled by the heterozygous load in tetraploid potato, it indicates that there is an evolutionary signal distinct from the mean effect of the derived alleles at the constrained sites that is relevant for the selection of starting material.

Methodological differences may also contribute to the relatively low increase in prediction accuracy obtained by including evolutionary information in tetraploids. The MASPOT panel was genotyped using GBS, resulting in lower genome coverage and potentially reduced accuracy of genotype calling and imputation compared to the diploid study. Improved genotyping resolution could refine estimates of allele dosage, deleterious and hypervariable loads, and, in turn, potentially enhance their effect on prediction performance – especially the genotyping of heterozygotes, which requires high read depth in tetrasomic polyploids in general (60-80x) [35], and is further complicated by extensive gene copy number variation across the tetraploid potato genome [36–39]. To minimize the effect of inaccurate heterozygous allele dosage calls, we calculated the heterozygous loads assuming non-additivity. Only the genetic loads relied on the discrete allele dosage, and they were not used beyond correlation analyses. More accurate variant effect prediction could also improve load estimation. For example, including an MSA at smaller evolutionary timescales for GERP scoring, e.g., using pangenomes in potato or in *Solanum* [29,65], may better capture functionally relevant genetic variation compared to a deep phylogeny. Additionally, DNA large language models may offer higher accuracy of variant effect prediction by incorporating contextual features such as codon usage, regulatory motifs, and gene structure [66–70]

Lastly, diploid breeding systems, particularly those leveraging complementary heterotic groups, are expected to retain greater potential gains from incorporating load-based predictors in genomic prediction compared to a tetraploid population based on genetically complex founders. Evaluating the predictive value of hypervariable load in diploid breeding systems remains an important direction for future research. Similarly, the evaluation of traits with disparate heritabilities and genetic architecture (i.a. polygenic, dominant, etc.) in diploid panels is also of interest, as both our results and those from showed a trait-specific response to the incorporation of the homozygous mutational loads [28].

### 3.4 Hypotheses for the selection of the derived allele at hypervariable sites in potato

We here consider two possible hypotheses for enrichment of the derived allele at hypervariable sites: 1) clade-specific positive selection driven by a beneficial contribution to organismal fitness and 2) balancing selection to maintain heterozygosity.

#### 3.4.1 Hypothesis 1: Clade-specific positive selection.

**Fig 6:**
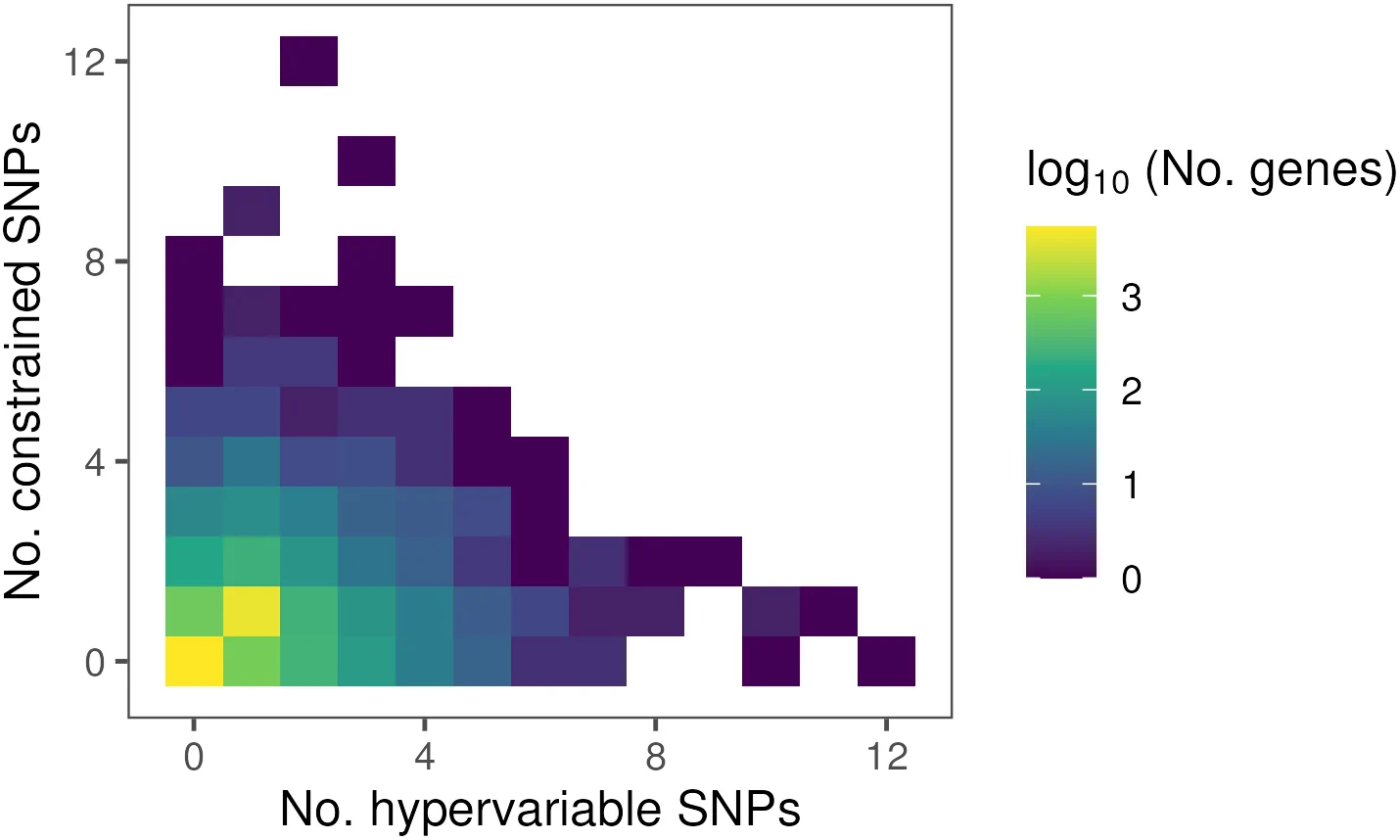
Several genes harbor both constrained and hypervariable sites. Heatmap of the number of hypervariable sites (GERP < -5.5) in a gene versus the number of constrained sites (GERP > 2.75) in the same gene in the MASPOT panel. The analysis includes all expressed genes in the DM v6.1 potato reference genome, which were annotated based on RNA sequencing results across multiple tissues [75].

Enrichment of the derived allele at hypervariable sites may reflect positive clade-specific selection in potato, which would explain both their enrichment in homozygosity and negative GERP scores (consistent with accelerated divergence above neutral expectations [33,71]). However, this also implies a fitness advantage of the enriched sSNPs. If not driven by MSA artifacts, this poses a conundrum as sSNPs are traditionally considered neutral, although this has recently been debated [72–74]. Allelic fitness depends on molecular and ecological context [6], and thus genomic constraint and hypervariability are likely to operate on multiple scales, from genomic regions to single codons [71]. This is consistent with several genes harboring both types of genetic variation (Fig 6) - however, limited SNP sampling might confound this pattern. While it could be beneficial for some genes, e.g., with functional redundancy in gene families, to be diversified by hypervariability to facilitate neofunctionalization or subfunctionalization, only single positions in other genes might allow this.

Signatures of clade-specific selection for hypervariability could be examined using phenology traits [76], e.g., tuberization in potatoes. It would also be highly relevant to explore the drivers of genomic hypervariability, and constraint [3,22], across evolution, domestication, and modern breeding in the *Solanaceae* family. If under positive selection, the hypervariable sites may represent a valuable source of genetic diversity for breeding. However, background linkage within haplotypes might greatly complicate the application of disparate breeding directions for these two types of genetic variation, i.e., purging the deleterious load while accumulating the potentially beneficial alleles at hypervariable sites [77], as linkage decay extends in the range of 0.6 Mb in modern tetraploid potato cultivars [78].

#### 3.4.2 Hypothesis 2: Balancing selection.

Accumulation of hypervariability could also reflect balancing selection maintaining heterozygosity [79]. Overdominance through heterozygote advantage is consistent with the complementation pattern of the hypervariable sites. However, the effect of balancing selection only becomes large with low mutation rates and very strong selection. In bottlenecked breeding populations of low effective size, genetic drift likely limits the maintenance of heterozygosity by balancing selection [80], except at sites under intense selection (e.g., resistance or self-incompatibility genes [79,81]). Comparing the drivers of selection pre- and post-modern breeding may clarify whether balancing selection was sufficiently strong to drive hypervariability. The limited genome coverage of MASPOT genotypes restricts assessment of gene-level enrichment for heterozygosity at hypervariable sites, but higher genotype density panels, e.g., the landrace panel of Wu et al., (2023), would enable tests for positive or balancing selection signatures predating the modern breeding bottleneck in hypervariable regions, e.g., using Tajima’s D [82–84]. However, the enrichment of synonymous variation at hypervariable sites may bias selection metrics such as dN/dS [85] and inflate signals of purifying selection in these regions, underscoring the need to clarify the origins and propagation of hypervariability.

## 4 Conclusions

The genome-wide inference of evolutionarily constrained sites in the potato genome constitutes a valuable resource for studies of potato genetic diversity and for future breeding strategies. As previously demonstrated, the incorporation of the deleterious mutation load can be used to facilitate the generation of inbred diploid lines for hybrid potato breeding and the subsequent purging of the deleterious load through designed crosses. Our results are consistent with this significant negative correlation between deleterious genetic load and homozygous load, indicating that individuals with the lowest deleterious load also have the lowest heterozygous load and the highest homozygous load. Our results also revealed significant correlation between deleterious load and yield, dry matter content, flesh color, and tuber count. More importantly, our study indicated that even though deleterious load in tetraploid potatoes is more complex than in diploid potato, quantitative genetics analysis benefitted from this evolutionary approach for tuber yield only (5% increased prediction accuracy with hypervariable loads), possibly due to the tetrasomic inheritance, a higher proportion of heterozygosity compared to homozygosity in tetraploid compared to diploid potato, and/or our GBS genotyping assay. However, phylogenomic mapping and estimation of the deleterious mutational load in the tetraploid potato genome still appears highly relevant for breeding, and the detailed mapping of the deleterious load constitutes a viable approach to purging in tetraploid potato, e.g., by genome editing. Lastly, our analysis indicated that significant information might be gleaned from the identification of hypervariable sites in the potato genome, as this genetic variation might point to beneficial alleles relevant for breeding. Further elucidation of this type of genetic variation is warranted. It is of particular interest to explore their effect on diploid genomic prediction models and whether they can contribute insight into evolutionary selection pressures for allelic diversification.

## 5 Methods

All statistical analyses and plotting were performed in R Statistical Software (v4.4.0) [86] in RStudio (v2024.04.2 + 764) [87] using custom scripts. Graphics were generated using base R or the ggplot2 package (v3.5.1) in R [88] unless otherwise specified.

### 5.1 Plant material

The MASPOT panel was created at Danespo A/S and is a representative 768 clone subset of the full MASPOT F_1_ population of phenotyped 5,013 clones from an incomplete diallel cross of 18 parents. The parent materials were either commercial cultivars or elite breeding clones of the Danespo A/S cultigen pool and were selected to share at most 12.5% relationship through pedigree. The population was generated by systematic cross-pollination of all parents in both cross-directions. F_1_ offspring were only selected for viable plant generation and tuberization properties (three tubers/plant) required for propagation. The diallel was thus only inhibited by male sterility of five cultivars and infertility of specific crosses in addition to plant degeneration or non-tuberization properties. The 5,013 phenotyped clones passed these selection criteria. The diallel crossing scheme of the MASPOT panel is available in [43] and of the full phenotyped MASPOT population in [40].

The MASPOT panel was planted from tuber seedlings in field trials in Vandel, Denmark in 2013 and 2014, as previously described [43,89]. The plant materials were planted in 24-parcel blocks in 2013 (no replicates, no checks) and in randomized 28-parcel blocks in 2014 (two replicates, 19 checks in two replicates, including the MASPOT parents). As propagation was concurrent with phenotyping trials, fewer tubers were available in 2013 than in 2014, and no formally established block design could be applied. Hence, we cannot mitigate any confounding of spatial effects with genotypic effects on individual phenotypic values, which might result in some systematic bias of the BLUEs and some loss of statistical power. However, as our main purpose was to maximize the genetic diversity trialed each year, including the clones performing close to the minimum viability/tuberization selection thresholds was prioritized to the minimize any survivor bias imposed through too strict selection criteria, which would subtract “low performers”, e.g., clones with high realized genetic loads, and bias prediction model training sets. Plant density was approximately 40,000 plants/ha, and plants were spaced with 30 cm between individual plants and 75 cm between rows. The clones were grouped according to senescence in 2014, based on preliminary phenotype data from 2013, to reduce neighbor vigor effects in the manual phenotyping trials. Only the 2014 senescence phenotypes are included in this study.

### 5.2 Phenotyping

Phenotypes were evaluated for eight traits: dry matter content, yield, senescence, flesh color, tuber count, and three tuber size measures, i.e., length, diameter, and volume (size). Dry matter content was evaluated in both 2013 and 2014, whereas the remaining phenotypes were only evaluated in 2014.

The dry matter content [%] was determined using an empirical modification of the Haase, (2003) [90] equation for dry matter content estimation from specific gravity based on best linear fit with Danespo A/S data (Equation 1):


$$\begin{array}{c} DM[\%]=\text{214}\cdot \left(\left(\frac{weight\;in\;air}{\left(weight\;in\;air)-(weight\;in\;water\right)}\right)-\text{0}.\text{988}\right) \end{array}$$ (1)

Tubers were washed, and a basket holding 1.5-10 kg tubers of each plant was weighed above and below water shortly after harvest.

The total yield of a plant was measured as the total weight of five tubers from each clone in two replicates, i.e., 2x5 tubers each. The weight was then converted into hkg/ha units based on the 40,000 plants/ha plant density. The tuber count was determined as the total number of tubers per clone.

Plant senescence was assessed by manual grading in the field using a scale of 1–9 from no senescence to late senescence. The scoring was performed at three temporal points using subsets of the scale: 9-8-7 for the first scoring when the first clones begin to die off, 6-5-4 at the second scoring, and 3-2-1 at the last scoring that captures the late senescing clones. Each clone was only scored once. Preliminary scoring was performed in 2013 and was used to guide the 2014 field layout according to senescence.

Tuber flesh color was graded manually on a scale of 1–9, from white to orange.

Whole-tuber length and diameter were measured using a SCOUT camera (Nextec A/S, no.: 0213). Tuber length was defined as the longest measure [mm] and diameter as the perpendicular measure to this [mm]. These measures did not consider tuber anatomy, where length describes the distance from the rose (apex) to the heel (attachment of stolon). This is generally the longest tuber measure, and only irregular tubers will violate this rule. These were assumed to be rare and minimally impact downstream analyses. Outliers were identified on both the length and diameter measures relative to the nearest neighbor and removed following the Dixon’s Q-test [91]. The critical confidence level (Q_crit_) was estimated for batches of up to 200 tubers by regression and used for a two-tailed test as described in Rorabacher, (1991) [92]. Following outlier filtration, tuber size (i.e., volume) was computed from length and diameter measures using Equation 2 for the volume of a prolate ellipsoid:


$$\begin{array}{c} Size\left[mm^{\text{3}}\right]=\frac{\text{4}}{\text{3}}\cdot \pi \cdot {\left(\frac{Diameter\;[mm]}{\text{2}}\right)}^{\text{2}}\cdot \frac{Length[mm]}{\text{2}} \end{array}$$ (2)

### 5.3 Genotyping of MASPOT panel

Genotyping of the MASPOT panel was performed by GBS. GBS libraries were prepared according to the descriptions in Sverrisdóttir et al., (2017) [89], following a protocol adapted from Elshire et al., (2011) [93]. The 5’ and 3’ Illumina sequencing adapters were designed for a 96-multiplex system. DNA was extracted from leaf tissue and subsequently digested with *ApeKI* restriction enzyme. The fragments were ligated to adapters and pooled in 96-plex libraries. The libraries were purified and amplified by PCR and subsequently sequenced on a HiSeq 2000 (Illumina, San Diego, CA, USA) with single-read sequencing (100 bp). Each 96-plex library was sequenced on three channels of a flow cell.

The sequenced reads were demultiplexed and trimmed as described in Sverrisdóttir et al., (2017) [89]. The reads were then mapped onto the doubled monoploid *S. tuberosum* Group Phureja reference genome sequence (DM v6.1 [94]) using BWA-MEM (v0.7.18) [95]. The mapped reads were sorted using samtools (v1.21) [96]. Base quality score recalibration (BQSR) was performed using the Genome Analysis Toolkit (GATK v4.5.0.0) [97,98]. Recalibration tables were generated using known variant sites derived from whole-genome sequencing of the parental lines. The recalibration was then applied to the BAM files before variant calling. Variants were called using GATK’s HaplotypeCaller in GVCF mode with a ploidy setting of four. GVCFs were merged using GATK’s CombineGVCFs, followed by joint genotyping with GenotypeGVCFs. SNPs and INDELs were filtered with a Root Mean Square (RMS) mapping quality threshold of at least 30, and only biallelic variants were retained. Minor allele frequency (MAF) was calculated from read coverage, and SNPs were filtered to retain only those with a minimum MAF of 1% (average variant allele frequency between 0.01 and 0.99). Additional filtering criteria included read coverage between 5x for each genotype call, mean variant read coverage < 60x, and a maximum missing data rate of 50%. All processing steps were implemented within a Snakemake [99] workflow to ensure reproducibility and computational efficiency.

This created a SNP set of 151,164 genomic variants that also had GERP scores (see 5.4) for 768 clones. The SNP density across the genome was plotted using the CMplot package (v4.5.1) [100] (**S36 Fig**). Imputation was performed using a random forest approach with 50 trees using the missRanger R package (v2.6.1) [101]. A total of 4.73% of the entries were imputed. The imputation accuracy was assessed as the Pearson correlation between genotypes imputed after artificially increasing missingness and genotypes from the original dataset without additional masking. Based on the observed missing rate, proportions of 3% and 7% artificially missing values were introduced randomly in eight repeats, yielding mean imputation accuracies of 0.884 (s.d. = 0.0006) and 0.882 (s.d. = 0.0004) for the 3% and 7% missing levels, respectively (**S9 Table**).

### 5.4 Phylogenomics and GERP++ scoring

The phylogenomics and GERP++ scores utilized in this study originated from the results of [28]. GERP++ scores were reduced to the filtered set of MASPOT panel SNPs. To ensure sufficient alignment depth to identify the homozygous burden, a filter of alignment depth ≥ 50 and neutral score ≥ 2 was used in load calculations, reducing the MASPOT panel SNP set to 97,815 variants with high quality GERP scores.

### 5.5 Relationship between derived allele frequency and GERP scores

The non-linear relationship between the derived allele (the minor allele of the 100-*Solanaceae* multiple-genome alignment from [28]) frequency (DAF) of the MASPOT panel and the GERP scores across the potato genome was estimated using a generalized additive model. The analysis was performed using the gam() function of the mgcv package (v1.9-1) [102].

### 5.6 Identification of deleterious and hypervariable variants

The results presented in the manuscript mainly present the moderately (2.75 ≤ GERP < 3.5) and highly constrained sites (GERP ≥ 3.5), as well as the moderately (-5.5 ≥ GERP > -7) and highly hypervariable sites (GERP ≤ -7). Mildly constrained (2 ≤ GERP < 2.75) and hypervariable (-4 ≥ GERP > -5.5) sites were also annotated, as well as remaining sites with intermediate GERP score (-4 < GERP < 2). A total of 78.33% of the minor allele variants in the MASPOT panel coincided with the minor alleles of the 100-*Solanaceae* genome alignment, suggesting that the evolutionarily derived mutations conform well with the minor alleles observed in a sexual cross of elite breeding clones.

Mapping of constrained and hypervariable sites to expressed genes in the DM v6.1 potato reference genome [94] used RNA sequencing data from seven tissues of E8669 and eight tissues of PG6359 [75] to annotate 9,853 pseudogenes in the reference genome (S3 Material) as those with mRNA expression levels of < 0.5 across all tissues.

### 5.7 Estimation of deleterious and hypervariable mutation loads

The genome-wide deleterious and hypervariable loads for each clone were calculated by summing the GERP scores of all inferred constrained or hypervariable sites. The loads were partitioned into a homozygous load by summing the genome-wide GERP scores of inferred constrained or hypervariable sites with homozygous genotypes (genotype = BBBB, with the derived allele (B) defined as the minor allele of the 100-*Solanaceae* genome alignment) (Equation 3). Analogously, the heterozygous loads were calculated for each of the heterozygous states by summing the GERP scores of inferred constrained or hypervariable sites with genotypes in that particular heterozygous state, and a combined heterozygous load was calculated by summing the GERP scores across all heterozygous states (Equation 4).


$$\begin{array}{c} b_{homo}=\sum_{k=\text{1}}\delta_{k}m_{k} \end{array}$$ (3)

$$\begin{array}{c} b_{het(simplex)}=\sum_{k=\text{1}}\delta_{k}{n_{1}}_{k} \end{array}$$

$$\begin{array}{c} b_{het(duplex)}=\sum_{k=\text{1}}\delta_{k}{n_{2}}_{k} \end{array}$$

$$\begin{array}{c} b_{het(triplex)}=\sum_{k=\text{1}}\delta_{k}{n_{3}}_{k} \end{array}$$

$$\begin{array}{c} \;b_{het}=\sum_{k=\text{1}}\delta_{k}{n_{all}}_{k}=b_{het(simplex)}+b_{het(duplex)}+b_{het(triplex)} \end{array}$$ (4)

Where ***b*** is a vector of heterozygous or homozygous deleterious/hypervariable loads across individuals, $\delta_{k}$ is the GERP score of SNP *k*, $m_{k}$ is a presence/absence indicator of the inference of a constrained/hypervariable variant in a homozygous state at SNP *k* across individuals ($m_{k}$ = 1 for derived alleles at constrained/hypervariable sites, otherwise $m_{k}$ = 0), and ${n_{x}}_{k}$ are presence/absence indicators of the inference of a constrained/hypervariable variant in any heterozygous state at SNP *k* at any given individual (${n_{x}}_{k}$ = 1 for the derived allele at the constrained/hypervariable sites in dosage x [x=all denotes all heterozygous allele dosages, i.e., 1, 2, and 3], otherwise ${n_{x}}_{k}$ = 0).

The genetic deleterious and hypervariable loads for each clone were calculated following Bertorelle et al. (2022) [103] modified for tetraploidy [104], where the genetic load represents the deleterious or hypervariable load potentially transmitted to progeny and affecting progeny fitness (Equation 5). The heterozygous loads in each heterozygous state were weighted according to the probability of a derived allele being transmitted to offspring based on allele dosage, assuming no double reduction. For computation of load correlations, a GERP threshold of GERP > 2.75 was used to annotate evolutionarily constrained sites, and a GERP threshold of GERP < -5.5 was used to annotate hypervariable sites.


$$\begin{array}{c} b_{genetic}=b_{homo}+\text{0}.\text{2}\text{5}\cdot b_{het(simplex)}+\text{0}.\text{5}\cdot b_{het(duplex)}+\text{0}.\text{7}\text{5}\cdot b_{het(triplex)} \end{array}$$ (5)

Where $b_{genetic}$ is a vector of deleterious/hypervariable genetic loads across individuals.

Using an analogous approach, we computed genome-wide counts of derived alleles at homozygous or heterozygous states by summing instances of homozygous and heterozygous (AAAB, AABB, ABBB) states, respectively, across all genome-wide variants for each clone. A derived allele genetic load was then computed following Equation 5.

### 5.8 Heterozygosity, gene diversity, and inbreeding coefficient

The observed heterozygosity ($H_{O}$) was calculated for each SNP in each clone in the MASPOT panel as the gametic heterozygosity [105] (Equation 16), i.e., the frequency of heterozygotes among randomly sampled diploid gametes formed from the four allele copies present at a locus in a tetraploid [106].


$$\begin{array}{c} H_{O}=\frac{d\cdot (\text{4}-d)}{\text{6}} \end{array}$$ (16)

, where *d* is the derived allele dosages. The mean $H_{o}$ was then computed for each SNP across all clones.

The gene diversity (or Nei’s expected heterozygosity [$H_{S}$]) was calculated for each SNP in the MASPOT panel using Equation 17 for polyploids [106].


$$\begin{array}{c} H_{S}=\text{1}-\sum p_{i}^{\text{2}}, \end{array}$$ (17)

where $p_{i}$ is the frequency of the ith allele at a single locus.

Wright’s inbreeding coefficient ($F_{IS}$) was calculated for each SNP in the MASPOT panel to quantify the departure from Hardy-Weinberg equilibrium using Equation 18:


$$\begin{array}{c} F_{IS}=(H_{S}-H_{O})/H_{S} \end{array}$$ (18)

The mean and standard deviation of the inbreeding coefficient across all SNPs was weighted by the expected heterozygosity (Equation 19), where i indicates the ith SNP:


$$\begin{array}{c} mean\left(F_{IS}\right)=\frac{\sum {F_{IS}}_{i}\cdot {H_{S}}_{i}}{\sum {H_{S}}_{i}},sd\left(F_{IS}\right)=\sqrt{\frac{\sum {H_{S}}_{i}\cdot {\left({F_{IS}}_{i}-mean\left(F_{SI}\right)\right)}^{\text{2}}}{\sum {H_{S}}_{i}}} \end{array}$$ (19)

Linear regressions between these three population statistics $H_{O}$, $H_{S}$, and $F_{IS}$ as a function of SNP GERP score was performed using the *lm()* function in R, and general additive models utilized the *gam()* function of the mgcv (v1.9-1) R package [102].

### 5.9 Genomic prediction

For stringency in genomic prediction modeling, we regarded alleles at sites with GERP scores > 3.5 as potentially deleterious and GERP < -7 as potentially hypervariable when computing the loads (Equations 3–4), considering the minor allele of the 100-*Solanaceae* genome alignment (the derived allele) as the potentially deleterious/hypervariable allele. A baseline genomic prediction model was used to benchmark the genomic prediction accuracy of the traits. The applied model was a GBLUP-based linear-mixed model (Equation 7):


$$\begin{array}{c} y=\mu +Xa+Z_{u}u+e \end{array}$$

$$\begin{array}{c} u\;\sim \;N(\text{0},G\sigma_{u}^{\text{2}}) \end{array}$$

$$\begin{array}{c} G=\frac{ZZ^{\prime }}{\text{4}\sum p_{k}(\text{1}-p_{k})\;} \end{array}$$ (7)

Here, ***y*** is a vector of recorded phenotypic values; µ is the grand mean; ***X*** is a design matrix for the fixed effects and ***a*** is a vectorof fixed effects, including year-by-block; $Z_{u}$ is an incidence matrix of the F_1_ clones, ***u*** is a vector of the additive genomic effects (breeding values), G is the genomic relationship matrix, and $\sigma_{u}^{\text{2}}$ is the additive genetic variance; ***e*** is a vector of residuals with $e\;\sim \;N\left(\text{0},I\sigma_{e}^{\text{2}}\right)$ with identity matrix ***I*** of dimension number of observations, and $\sigma_{e}^{\text{2}}$ is the residual variance. The genomic relationship matrix was calculated using the VanRaden method 1 [107] with adjustment for tetraploidy, where ***Z*** is the centered genotype matrix of reference allele counts (0,1,2,3, or 4), and $\text{4}\sum p_{k}(\text{1}-p_{k})\;$ is the sum of genotypic variance, where $p_{k}$ is the mean allele frequency at SNP *k* (S37–S38 Figs).

In the deleterious and hypervariable load models, the effects of the genome-wide homozygous and heterozygous loads were estimated separately and combined as fixed effects and the additive genomic effects as a random effect (Equation 8).


$$\begin{array}{c} y=\mu +Xa+\alpha_{total}b_{total}+Z_{u}u+e \end{array}$$

$$\begin{array}{c} y=\mu +Xa+\alpha_{homo}b_{homo}+Z_{u}u+e \end{array}$$

$$\begin{array}{c} y=\mu +Xa+\alpha_{het}b_{het}+Z_{u}u+e \end{array}$$

$$\begin{array}{c} y=\mu +Xa+\alpha_{homo}b_{homo}+\alpha_{het}b_{het}+Z_{u}u+e \end{array}$$ (8)

Where $b_{homo}$ and $b_{het}$ are the genome-wide homozygous and heterozygous load (either deleterious or hypervariable) with coefficients $\alpha_{homo}$ and $\alpha_{het}$, respectively. To include a control for the effect of inbreeding, a model including a load of total derived alleles ($b_{total}=\sum_{k=\text{1}}q_{k}$, where *q* is the derived allele dosage) with coefficient $\alpha_{total}$ was also tested.

The genomic prediction models were fitted using the RAINBOWR (v0.1.35) [108] wrapper of the MM4LMM package (v3.0.3) [109] in R. The models were evaluated with leave-nine-families-out (LNFO) cross-validation (13 folds of random nine-family groups of the 117 families [reciprocals were included as the same family]), to minimize prediction inflation due to inclusion of full-sibs in training folds [110]. Cross-validation was performed in 20 repeats with alternative groupings in the folds in each repeat. Predictive ability was quantified as the Pearson correlations between observed and predicted phenotypes for each LNFO repeat. A corrected t-test for correlated samples was used for pairwise comparison prediction correlation coefficients of baseline and permuted load models with observed loads for each trait across cross-validation repeats. *p*-values were Bonferroni corrected for total number of comparisons for each trait with a significance threshold of 0.05. The correctR package (v0.1.3) was used to compute test statistics [111].

To verify whether including weighted mutation loads conferred additional information to genomic prediction models, a set of random SNP scores was generated by permuting GERP scores among windows of 1,000 SNPs to account for linkage disequilibrium among contiguous SNPs (the mean window length was 2,627,412 bp). A total of 100 permutations were generated, and for each permutation, the random SNP scores were used to compute genome-wide load indices, $b_{homo}$ and $b_{het}$, for both the deleterious and hypervariable loads. Then, the effect of the random SNP scores was estimated for each of the permutations in LNFO cross-validation as described above for each of the 100 permutations.

The narrow sense additive genetic plot heritability for each trait was computed as the ratio of additive genetic ($\sigma_{u}^{\text{2}}$) to total phenotypic variance ($\sigma_{p}^{\text{2}}$) (Equation 10):


$$\begin{array}{c} h^{\text{2}}=\frac{\sigma_{u}^{\text{2}}}{\sigma_{p}^{\text{2}}},\;\sigma_{p}^{\text{2}}=\sigma_{u}^{\text{2}}+\sigma_{l}^{\text{2}}+\sigma_{e}^{\text{2}} \end{array}$$ (10)

It was inferred based on the following model, separating the additive genetic variance from the non-additive genetic variance by including a random line effect to capture the effect of clone replicates: $\mu +Xb+Z_{u}u+Z_{u}l+e$, where l is a vector of line effects with $l\;\sim \;N(\text{0},I_{l}\sigma_{l}^{\text{2}})$, where $I_{l}$ is an identity matrix with the number of lines as dimensions.

### 5.10 Genome-wide association studies

For stringency, we regard derived alleles at sites with GERP scores > 3.5 as potentially deleterious and GERP < -7 as potentially hypervariable when computing the loads for GWAS as well (Equations 3–4). GWAS was performed for each trait using the GWASpoly package (v2.13) in R [112]. A baseline GWAS model was used to benchmark the GWAS performance for each of the traits. The applied model was a GBLUP-based linear-mixed model (Equation 11):


$$\begin{array}{c} y=\mu +Wc+X_{i}\beta_{i}+Z_{u}u+e \end{array}$$ (11)

Here, ***y*** is a vector of recorded phenotypic values; µ is the grand mean; ***W*** is a design matrix for the fixed effects and ***c*** is a matrix of fixed effects, including year-by-block; ***X***_***i***_ is the vector of SNP genotype at the ith position; *β*_*i*_ is the corresponding genetic effect of the ith SNP; $Z_{u}$ is an incidence matrix of the F_1_ clones, ***u*** is a vector of the additive genomic effects (breeding values) with $u\;\sim \;N(\text{0},G\sigma_{u}^{\text{2}})$, where G is the genomic relationship matrix and $\sigma_{u}^{\text{2}}$ is the additive genetic variance; ***e*** is a vector of residuals with $e\;\sim \;N\left(\text{0},I\sigma_{e}^{\text{2}}\right)$ with identity matrix ***I*** of dimension number of observations, and $\sigma_{e}^{\text{2}}$ is the residual variance. A new G-matrix was computed for each chromosome using the GWASpoly package with leave-one-chromosome-out (LOCO) association testing.

In the deleterious and hypervariable load GWAS models, the genome-wide homozygous and heterozygous loads were included separately as fixed effects and GWAS was again performed by additive single variant regression with LOCO association testing for each of the included loads (Equation 12):


$$\begin{array}{c} y=\mu +Xb+\alpha_{total}b_{total}+X_{i}\beta_{i}+Z_{u}u+e \end{array}$$

$$\begin{array}{c} y=\mu +Wc+\alpha_{homo}b_{homo}+X_{i}\beta_{i}+Z_{u}u+e \end{array}$$

$$\begin{array}{c} y=\mu +Wc+\alpha_{het}b_{het}+X_{i}\beta_{i}+Z_{u}u+e \end{array}$$ (12)

Where $b_{homo}$ and $b_{het}$ are the genome-wide homozygous and heterozygous load (either deleterious or hypervariable) with coefficients $\alpha_{homo}$ and $\alpha_{het}$, respectively. Again, a model incorporating a sum of derived alleles was also tested, where $b_{total}$ is the genome-wide load of derived alleles with coefficient $\alpha_{total}$, serving as a control for including the mean effect of derived allele inherently associated with the mutational loads.

To correct the *p*-values for overdispersion due to stratified populations (e.g., through inbreeding) [113], we used the genomic inflation factor $\lambda_{gc}$ [114]. Genome-wide genomic inflation factors were calculated for each model for each trait using Equation 13:


$$\begin{array}{c} \lambda_{gc}=\frac{median\left(\chi^{\text{2}}\right)}{Q_{\chi^{\text{2}}}^{-\text{1}}(\text{0}.\text{5},\text{1})}, \end{array}$$

$$\begin{array}{c} \chi^{\text{2}}=Q_{\chi^{\text{2}}}^{-\text{1}}(P,\text{1}) \end{array}$$ (13)

The genomic inflation factor, $\lambda_{gc}$ (S10 Table), was computed as the median value of the $\chi^{\text{2}}$-statistics of the SNPs, converted from each *p*-value, *P*, using the inverse cumulative distribution function (CDF) of the $\chi^{\text{2}}$-distribution, $Q_{\chi^{\text{2}}}^{-\text{1}}$, with 1 degree of freedom, divided by the expected median, assuming no association between the SNP and the trait phenotype, i.e., the $\chi^{\text{2}}$-statistic of the 50^th^ percentile. For models with $\lambda_{gc}$ > 1, indicating systematic effects not captured by the model, a correction was performed by dividing the SNP $\chi^{\text{2}}$-statistics by $\lambda_{gc}$ and the resulting values were converted to corrected *p*-values using the CDF of the $\chi^{\text{2}}$-distribution with 1 degree of freedom (Equation 14):


$$\begin{array}{c} P_{corrected}=\text{1}-Q_{\chi^{\text{2}}\;}\left(\left(\frac{\chi^{\text{2}}}{\lambda_{gc}}\right),\text{1}\right) \end{array}$$ (14)

Bonferroni correction was used to control false positive associations with a false discovery rate of *p* < 0.05/N, where 0.05 is the significance threshold and N is the number of variants tested. QQ and Manhattan plots were generated using the qqman package (v0.1.9) [115].

The genetic variance explained by the most significant SNP of each QTL was calculated using Equation 15 [116] with correction for tetraploidy:


$$\begin{array}{c} \sigma_{SNP_{i}}^{\text{2}}=\text{4}\cdot \beta_{i}^{\text{2}}\cdot f_{i}(\text{1}-f_{i}) \end{array}$$ (15)

, where $\beta_{i}$ is the effect size and $f_{i}$ is the MAF of the ith SNP. This was used to calculate the fraction of phenotypic and additive genetic variance explained by each of the most significant QTL SNPs using the total phenotypic ($\sigma_{p}^{\text{2}}$) and genetic variances ($\sigma_{g}^{\text{2}}$) explained by the model.

## Supporting information

S1 Fig Genotype variance is significantly associated with GERP score. Association between the genotype variance at each locus and GERP fitted using a generalized additive model. The second axis shows the effect of GERP on genotype variance.
(DOCX)

S2 Fig GERP score is significantly associated with minor allele frequency in the landrace panel. Association between the minor allele frequency in the (Wu et al., 2023) landrace panel for 6,850,272 SNPs with multiple sequence alignment depth > 50 and neutral score > 2 fitted using a general additive model.
(DOCX)

S3 Fig GERP score density. Density plot of GERP scores of the final 97,815 SNP set.
(DOCX)

S4 Fig Proportions of constrained and hypervariable markers. Distribution of variants according to GERP threshold in the landrace panel for markers with confidently assigned GERP scores (neutral > 2 and depth > 50), i.e., ~ 6 mio. markers. Number of variants in each group and percentage of all variants are shown above.
(DOCX)

S5 Fig Homozygous and heterozygous mutational loads strongly correlate with overall clone homozygosity and heterozygosity. Pearson correlations between homozygous mutational loads (deleterious and positive hypervariable) and overall clone homozygosity and between heterozygous mutational loads (deleterious and positive hypervariable) and overall clone heterozygosity with Pearson correlation coefficients. ***p < 0.0001 in Pearson correlation test.
(DOCX)

S6 Fig Hypervariable and deleterious mutational loads are highly correlated. Top: Correlation between homozygous loads (deleterious and positive hypervariable) with Pearson correlation coefficients. Bottom: Correlation between heterozygous loads (deleterious and positive hypervariable) with Pearson correlation coefficients. ***p < 0.0001 in Pearson correlation test.
(DOCX)

S7 Fig Genome-wide derived allele loads reproduce trends in loads correlations found for deleterious and hypervariable loads. A) Density plots of derived allele loads and correlations among genetic, heterozygous, and homozygous derived allele loads. Pearson correlation coefficients among the loads are shown. B) The same plots and statistics, including clone F1-12-376-02. ***p < 0.0001 in Pearson correlation test.
(DOCX)

S8 Fig Deleterious load correlations including an outlier clone. Density plots of deleterious loads and correlations among genetic, heterozygous, and homozygous deleterious loads, including clone F1-12-376-02. Correlation coefficients among the loads are shown. *** *p* < 0.0001 in Pearson correlation test.
(DOCX)

S9 Fig Hypervariable load correlations including an outlier clone. Density plots of (positive) hypervariable loads and correlations among genetic, heterozygous, and homozygous hypervariable loads, including clone F1-12-376-02. Correlation coefficients among the loads are shown. *** *p* < 0.0001 in Pearson correlation test.
(DOCX)

S10 Fig Observed and expected heterozygosity are significantly associated with GERP score, following a linear trend. General additive model of A) observed heterozygosity (H_O_) in the MASPOT panel and B) expected heterozygosity (H_S_) in the MASPOT panel as a function of GERP score. The second axes show the effects of GERP score on H_O_ and H_S_, respectively.
(DOCX)

S11 Fig Observed heterozygosity was consistently lower than expected heterozygosity across all marker types. Mean observed heterozygosity (H_O_) [top] and gene diversity (expected heterozygosity [H_S_]) [bottom] across all grouped by marker annotation. The mean heterozygosity is shown as a black dot.
(DOCX)

S12 Fig Observed heterozygosity is significantly associated with GERP score, following a linear trend in the landrace panel. General additive model of observed heterozygosity (H_O_) in the landrace panel of Wu et al., (2023) as a function of GERP score. The observed heterozygosity was computed for 6,850,272 SNPs with multiple sequence alignment depth > 50 and neutral score > 2. The second axis show the effects of GERP score on H_O_.
(DOCX)

S13 Fig The inbreeding coefficient across had high variability across marker types. Mean observed inbreeding coefficient (F_IS_) across all markers (left), deleterious markers (center), and hypervariable markers (right) grouped by marker annotation. The mean inbreeding coefficient is shown as a black dot.
(DOCX)

S14 Fig Complementation of deleterious variants compared to non-deleterious mutations matched that compared to other mutations. Comparing the percentage of heterozygous genotypes (simplex-triplex) of non-deleterious mutations [GERP < 2] with that of mildly [2 ≤ GERP < 2.75], moderately [2.75 ≤ GERP < 3.5], and highly [GERP ≥ 3.5] deleterious mutations in the MASPOT panel. The dashed gray line indicates “*y = x*”. The solid lines represent linear regressions within groups of points and are shown with 95% confidence intervals in gray. Within each color group each point is one MASPOT F_1_ clone. (mild: t = 147.95, *p*-value < 2.2*10^-16^; moderate: t = 148.99, *p*-value < 2.2*10^-16^; high: t = 131.45, *p*-value < 2.2*10^-16^; paired Student’s t-test).
(DOCX)

S15 Fig The patterns of complementation of alleles at constrained and hypervariable loci were consistent for high-read depth genotypes alone. A) Comparing the percentage of heterozygous genotypes (simplex-triplex) of other mutations [-4 < GERP < 2] with that of mildly [2 ≤ GERP < 2.75], moderately [2.75 ≤ GERP < 3.5], and highly [GERP ≥ 3.5] deleterious mutations in the MASPOT panel for markers filtered for read depth > 20. (mild: t = 84.89, *p*-value < 2.2*10^-16^; moderate: t = 89.07, *p*-value < 2.2*10^-16^; high: t = 93.87, *p*-value < 2.2*10^-16^; paired Student’s t-test). B) The same comparison for mildly [-5.5 < GERP ≤ -4], moderately [-7 < GERP ≤ -5.5], and highly [GERP ≤ -7] hypervariable variants. (mild: t = -114.35, *p*-value < 2.2*10^-16^; moderate: t = -103.66, *p*-value < 2.2*10^-16^; high: t = -101.98, *p*-value < 2.2*10^-16^; paired Student’s t-test) The dashed gray line indicates “*y = x*”. The solid lines represent linear regressions within groups of points and are shown with 95% confidence intervals in gray. Within each color group each point is one MASPOT F_1_ clone.
(DOCX)

S16 Fig The ratio of homozygosity depreciates with GERP score at constrained loci and increases with GERP score at hypervariable loci. A) Comparing the percentage of homozygous genotypes and heterozygous genotypes (simplex-triplex) across mildly [2 ≤ GERP < 2.75], moderately [2.75 ≤ GERP < 3.5], and highly [GERP ≥ 3.5] deleterious mutations in the MASPOT panel. (mild: t = 301.3, *p*-value < 2.2*10^-16^; moderate: t = 310.38, *p*-value < 2.2*10^-16^; high: t = 276.17, *p*-value < 2.2*10^-16^, paired Student’s t-test). B) The same comparison for mildly [-5.5 < GERP ≤ -4], moderately [-7 < GERP ≤ -5.5], and highly [GERP ≤ -7] hypervariable variants. (mild: t = -345.53, *p*-value < 2.2*10^-16^; moderate: t = -315.58, *p*-value < 2.2*10^-16^; high: t = -303.33, *p*-value < 2.2*10^-16^, paired Student’s t-test). The solid lines represent linear regressions within groups of points and are shown with 95% confidence intervals in gray. Within each color group each point is one MASPOT F_1_ clone.
(DOCX)

S17 Fig Partial correlations between heterozygous deleterious load and phenotypes. Partial correlations between clone phenotype and the per-individual residual heterozygous deleterious load (after fitting the homozygous load and correcting for population structure [fitting principal components 1–3 of the genomic relationship matrix]) among the MASPOT panel clones for each trait. Pearson partial correlation coefficients and Pearson partial correlation test *p*-values are shown. The solid black line indicates the linear regression between phenotype and residual load. Gray shadows indicate 95% confidence intervals.
(DOCX)

S18 Fig Partial correlations between heterozygous hypervariable load and phenotypes. Partial correlations between clone phenotype and the per-individual residual positive heterozygous hypervariable load (after fitting the homozygous load and correcting for population structure [fitting principal components 1–3 of the genomic relationship matrix]) among the MASPOT panel clones for each trait. Pearson partial correlation coefficients and Pearson partial correlation test *p*-values are shown. The solid black line indicates the linear regression between phenotype and residual load. Gray shadows indicate 95% confidence intervals.
(DOCX)

S19 Fig Partial correlations between total derived allele load and phenotypes. Partial correlations between clone phenotype and the per-individual total count of derived alleles (after correcting for population structure [fitting principal components 1–3 of the genomic relationship matrix]) among the MASPOT panel clones for each trait. Pearson partial correlation coefficients and Pearson partial correlation test *p*-values are shown. The solid black line indicates the linear regression between phenotype and residual load. Gray shadows indicate 95% confidence intervals.
(DOCX)

S20 Fig Manhattan plots of GWAS of dry matter content. Manhattan plots of -log_10_(*p*-value) GWAS results of 6 models for dry matter content. From top to bottom: the baseline model, the total alternative allele model, the heterozygous deleterious load model, the homozygous deleterious load model, the heterozygous hypervariable load model, and the homozygous hypervariable load model. The significance threshold with Bonferroni correction is indicated with a horizontal line.
(DOCX)

S21 Fig Manhattan plots of GWAS of dry matter yield. Manhattan plots of -log_10_(*p*-value) GWAS results of 6 models for yield. From top to bottom: the baseline model, the total alternative allele model, the heterozygous deleterious load model, the homozygous deleterious load model, the heterozygous hypervariable load model, and the homozygous hypervariable load model. The significance threshold with Bonferroni correction is indicated with a horizontal line.
(DOCX)

S22 Fig Manhattan plots of GWAS of senescence. Manhattan plots of -log_10_(*p*-value) GWAS results of 6 models for senescence. From top to bottom: the baseline model, the total alternative allele model, the heterozygous deleterious load model, the homozygous deleterious load model, the heterozygous hypervariable load model, and the homozygous hypervariable load model. The significance threshold with Bonferroni correction is indicated with a horizontal line.
(DOCX)

S23 Fig Manhattan plots of GWAS of flesh color. Manhattan plots of -log_10_(*p*-value) GWAS results of 6 models for flesh color. From top to bottom: the baseline model, the total alternative allele model, the heterozygous deleterious load model, the homozygous deleterious load model, the heterozygous hypervariable load model, and the homozygous hypervariable load model. The significance threshold with Bonferroni correction is indicated with a horizontal line.
(DOCX)

S24 Fig Manhattan plots of GWAS of tubers/plant. Manhattan plots of -log_10_(*p*-value) GWAS results of 6 models for tubers/plant. From top to bottom: the baseline model, the total alternative allele model, the heterozygous deleterious load model, the homozygous deleterious load model, the heterozygous hypervariable load model, and the homozygous hypervariable load model. The significance threshold with Bonferroni correction is indicated with a horizontal line.
(DOCX)

S25 Fig Manhattan plots of GWAS of length. Manhattan plots of -log_10_(*p*-value) GWAS results of 6 models for tuber length. From top to bottom: the baseline model, the total alternative allele model, the heterozygous deleterious load model, the homozygous deleterious load model, the heterozygous hypervariable load model, and the homozygous hypervariable load model. The significance threshold with Bonferroni correction is indicated with a horizontal line.
(DOCX)

S26 Fig Manhattan plots of GWAS of diameter. Manhattan plots of -log_10_(*p*-value) GWAS results of 6 models for tuber diameter. From top to bottom: the baseline model, the total alternative allele model, the heterozygous deleterious load model, the homozygous deleterious load model, the heterozygous hypervariable load model, and the homozygous hypervariable load model. The significance threshold with Bonferroni correction is indicated with a horizontal line.
(DOCX)

S27 Fig Manhattan plots of GWAS of size. Manhattan plots of -log_10_(*p*-value) GWAS results of 6 models for tube size. From top to bottom: the baseline model, the total alternative allele model, the heterozygous deleterious load model, the homozygous deleterious load model, the heterozygous hypervariable load model, and the homozygous hypervariable load model. The significance threshold with Bonferroni correction is indicated with a horizontal line.
(DOCX)

S28 FigQQ plot comparison of GWAS models for dry matter content.QQ plots of -log_10_(*p*-value) GWAS results of the baseline model against [Expected *p*] (from left to right) -log_10_(*p*-value) GWAS results of the total alternative allele model (fitting the number of derived alleles), the homozygous deleterious load model (fitting the homozygous deleterious load), the heterozygous deleterious load model (fitting the heterozygous deleterious load), the homozygous hypervariable load model (fitting the homozygous hypervariable load),or the heterozygous hypervariable load model (fitting the heterozygous hypervariable load) [Observed *p*] for the trait dry matter content. The black line indicates x=y.(DOCX)

S29 FigQQ plot comparison of GWAS models for yield.QQ plots of -log_10_(*p*-value) GWAS results of the baseline model against [Expected *p*] (from left to right) -log_10_(*p*-value) GWAS results of the total alternative allele model (fitting the number of derived alleles), the homozygous deleterious load model (fitting the homozygous deleterious load), the heterozygous deleterious load model (fitting the heterozygous deleterious load), the homozygous hypervariable load model (fitting the homozygous hypervariable load),or the heterozygous hypervariable load model (fitting the heterozygous hypervariable load) [Observed *p*] for the trait yield. The black line indicates x=y.(DOCX)

S30 FigQQ plot comparison of GWAS models for senescence.QQ plots of -log_10_(*p*-value) GWAS results of the baseline model against [Expected *p*] (from left to right) -log_10_(*p*-value) GWAS results of the total alternative allele model (fitting the number of derived alleles), the homozygous deleterious load model (fitting the homozygous deleterious load), the heterozygous deleterious load model (fitting the heterozygous deleterious load), the homozygous hypervariable load model (fitting the homozygous hypervariable load),or the heterozygous hypervariable load model (fitting the heterozygous hypervariable load) [Observed *p*] for the trait senescence. The black line indicates x=y.(DOCX)

S31 FigQQ plot comparison of GWAS models for flesh color.QQ plots of -log_10_(*p*-value) GWAS results of the baseline model against [Expected *p*] (from left to right) -log_10_(*p*-value) GWAS results of the total alternative allele model (fitting the number of derived alleles), the homozygous deleterious load model (fitting the homozygous deleterious load), the heterozygous deleterious load model (fitting the heterozygous deleterious load), the homozygous hypervariable load model (fitting the homozygous hypervariable load),or the heterozygous hypervariable load model (fitting the heterozygous hypervariable load) [Observed *p*] for the trait flesh color. The black line indicates x=y.(DOCX)

S32 FigQQ plot comparison of GWAS models for tubers/plant.QQ plots of -log_10_(*p*-value) GWAS results of the baseline model against [Expected *p*] (from left to right) -log_10_(*p*-value) GWAS results of the total alternative allele model (fitting the number of derived alleles), the homozygous deleterious load model (fitting the homozygous deleterious load), the heterozygous deleterious load model (fitting the heterozygous deleterious load), the homozygous hypervariable load model (fitting the homozygous hypervariable load),or the heterozygous hypervariable load model (fitting the heterozygous hypervariable load) [Observed *p*] for the trait tubers/plant. The black line indicates x=y.(DOCX)

S33 FigQQ plot comparison of GWAS models for length.QQ plots of -log_10_(*p*-value) GWAS results of the baseline model against [Expected *p*] (from left to right) -log_10_(*p*-value) GWAS results of the total alternative allele model (fitting the number of derived alleles), the homozygous deleterious load model (fitting the homozygous deleterious load), the heterozygous deleterious load model (fitting the heterozygous deleterious load), the homozygous hypervariable load model (fitting the homozygous hypervariable load),or the heterozygous hypervariable load model (fitting the heterozygous hypervariable load) [Observed *p*] for the trait tuber length. The black line indicates x=y.(DOCX)

S34 FigQQ plot comparison of GWAS models for diameter.QQ plots of -log_10_(*p*-value) GWAS results of the baseline model against [Expected *p*] (from left to right) -log_10_(*p*-value) GWAS results of the total alternative allele model (fitting the number of derived alleles), the homozygous deleterious load model (fitting the homozygous deleterious load), the heterozygous deleterious load model (fitting the heterozygous deleterious load), the homozygous hypervariable load model (fitting the homozygous hypervariable load),or the heterozygous hypervariable load model (fitting the heterozygous hypervariable load) [Observed *p*] for the trait tuber diameter. The black line indicates x=y.(DOCX)

S35 FigQQ plot comparison of GWAS models for size.QQ plots of -log_10_(*p*-value) GWAS results of the baseline model against [Expected *p*] (from left to right) -log_10_(*p*-value) GWAS results of the total alternative allele model (fitting the number of derived alleles), the homozygous deleterious load model (fitting the homozygous deleterious load), the heterozygous deleterious load model (fitting the heterozygous deleterious load), the homozygous hypervariable load model (fitting the homozygous hypervariable load),or the heterozygous hypervariable load model (fitting the heterozygous hypervariable load) [Observed *p*] for the trait tuber size. The black line indicates x=y.(DOCX)

S36 Fig SNP density of the MASPOT panel follows genome-wide gene density. Heatmap of marker density in 1 Mb windows for each of the 12 chromosomes. The color gradient denotes window marker count.
(DOCX)

S37 Fig Principal component analysis of the genomic relationship matrix showed some family structure. Principal component analysis of the genomic relationship matrix colored by mother (left) and father (right). Principal component 1 is plotted against principal component 2 in the top and against principal component 3 in the bottom. Principal components 1–3 explain 20.3%, 11.06%, and 9.99% of the total explained variance, respectively.
(DOCX)

S38 Fig Heatmap of the genomic relationship matrix. (DOCX)

S1 Table Genomic prediction covariate coefficients for dry matter content. (DOCX)

S2 Table Genomic prediction covariate coefficients for yield. (DOCX)

S3 Table Genomic prediction covariate coefficients for senescence. (DOCX)

S4 Table Genomic prediction covariate coefficients for flesh color. (DOCX)

S5 Table Genomic prediction covariate coefficients for tubers/plant. (DOCX)

S6 Table Genomic prediction covariate coefficients for length. (DOCX)

S7 Table Genomic prediction covariate coefficients for diameter. (DOCX)

S8 Table Genomic prediction covariate coefficients for size. (DOCX)

S9 Table Imputation accuracies with 3% and 7% introduced missingness. (DOCX)

S10 Table Genomic inflation factors for GWAS of each trait. (DOCX)

S1 Material GWAS QTLs of each trait. (XLSX)

S2 Material GWAS QTL effect sizes and SNP heritabilities for each trait. (XLSX)

S3 Material Pseudogene list relative to the DM v6.1 potato reference genome based on RNA expression data. (XLSX)
