# Supplementary material for: Genomic constraint and hypervariability correlate with fitness effects in elite tetraploid potato breeding material
Source: PLoS Genet. 2026 Aug 11;22(8):e1012271. doi: 10.1371/journal.pgen.1012271 (PMC13492815; doi:10.1371/journal.pgen.1012271)
Supplement: Supplementary file 1 [file pgen.1012271.s001.docx]

**S1 Fig. Genotype variance is significantly associated with GERP score.** Association between the genotype variance at each locus and GERP fitted using a generalized additive model. The second axis shows the effect of GERP on genotype variance.
