# Supplementary material for: Genomic constraint and hypervariability correlate with fitness effects in elite tetraploid potato breeding material
Source: PLoS Genet. 2026 Aug 11;22(8):e1012271. doi: 10.1371/journal.pgen.1012271 (PMC13492815; doi:10.1371/journal.pgen.1012271)
Supplement: Supplementary file 2 [file pgen.1012271.s002.docx]

**S2 Fig. GERP score is significantly associated with minor allele frequency in the landrace panel.** Association between the minor allele frequency in the (Wu et al., 2023) landrace panel for 6,850,272 SNPs with multiple sequence alignment depth > 50 and neutral score > 2 fitted using a general additive model.
