# Supplementary material for: Genomic constraint and hypervariability correlate with fitness effects in elite tetraploid potato breeding material
Source: PLoS Genet. 2026 Aug 11;22(8):e1012271. doi: 10.1371/journal.pgen.1012271 (PMC13492815; doi:10.1371/journal.pgen.1012271)
Supplement: Supplementary file 4 [file pgen.1012271.s004.docx]

**S4 Fig. Proportions of constrained and hypervariable markers.** Distribution of variants according to GERP threshold in the landrace panel for markers with confidently assigned GERP scores (neutral > 2 and depth > 50), i.e. ~6 mio. markers. Number of variants in each group and percentage of all variants are shown above.
