# Supplementary material for: Genomic constraint and hypervariability correlate with fitness effects in elite tetraploid potato breeding material
Source: PLoS Genet. 2026 Aug 11;22(8):e1012271. doi: 10.1371/journal.pgen.1012271 (PMC13492815; doi:10.1371/journal.pgen.1012271)
Supplement: Supplementary file 5 [file pgen.1012271.s005.docx]

**S5 Fig. Homozygous and heterozygous mutational loads strongly correlate with overall clone homozygosity and heterozygosity.** Pearson correlations between homozygous mutational loads (deleterious and positive hypervariable) and overall clone homozygosity and between heterozygous mutational loads (deleterious and positive hypervariable) and overall clone heterozygosity with Pearson correlation coefficients. ****p* < 0.0001 in Pearson correlation test.
