# Supplementary material for: Genomic constraint and hypervariability correlate with fitness effects in elite tetraploid potato breeding material
Source: PLoS Genet. 2026 Aug 11;22(8):e1012271. doi: 10.1371/journal.pgen.1012271 (PMC13492815; doi:10.1371/journal.pgen.1012271)
Supplement: Supplementary file 6 [file pgen.1012271.s006.docx]

**S6 Fig. Hypervariable and deleterious mutational loads are highly correlated.** Top: Correlation between homozygous loads (deleterious and positive hypervariable) with Pearson correlation coefficients. Bottom: Correlation between heterozygous loads (deleterious and positive hypervariable) with Pearson correlation coefficients. ****p* < 0.0001 in Pearson correlation test.
