# Supplementary material for: Genomic constraint and hypervariability correlate with fitness effects in elite tetraploid potato breeding material
Source: PLoS Genet. 2026 Aug 11;22(8):e1012271. doi: 10.1371/journal.pgen.1012271 (PMC13492815; doi:10.1371/journal.pgen.1012271)
Supplement: Supplementary file 7 [file pgen.1012271.s007.docx]

**S7 Fig. Genome-wide derived allele loads reproduce trends in loads correlations found for deleterious and hypervariable loads. A)** Density plots of derived allele loads and correlations among genetic, heterozygous, and homozygous derived allele loads. Pearson correlation coefficients among the loads are shown. **B)** The same plots and statistics, including clone F1-12-376-02. ****p* < 0.0001 in Pearson correlation test.
