# Supplementary material for: Genomic constraint and hypervariability correlate with fitness effects in elite tetraploid potato breeding material
Source: PLoS Genet. 2026 Aug 11;22(8):e1012271. doi: 10.1371/journal.pgen.1012271 (PMC13492815; doi:10.1371/journal.pgen.1012271)
Supplement: Supplementary file 8 [file pgen.1012271.s008.docx]

**S8 Fig. Deleterious load correlations including an outlier clone.** Density plots of deleterious loads and correlations among genetic, heterozygous, and homozygous deleterious loads, including clone F1-12-376-02. Correlation coefficients among the loads are shown. *** *p* < 0.0001 in Pearson correlation test.
