# Supplementary material for: Genomic constraint and hypervariability correlate with fitness effects in elite tetraploid potato breeding material
Source: PLoS Genet. 2026 Aug 11;22(8):e1012271. doi: 10.1371/journal.pgen.1012271 (PMC13492815; doi:10.1371/journal.pgen.1012271)
Supplement: Supplementary file 9 [file pgen.1012271.s009.docx]

**S9 Fig. Hypervariable load correlations including an outlier clone.** Density plots of (positive) hypervariable loads and correlations among genetic, heterozygous, and homozygous hypervariable loads, including clone F1-12-376-02. Correlation coefficients among the loads are shown. *** *p* < 0.0001 in Pearson correlation test.
