# Supplementary material for: Genomic constraint and hypervariability correlate with fitness effects in elite tetraploid potato breeding material
Source: PLoS Genet. 2026 Aug 11;22(8):e1012271. doi: 10.1371/journal.pgen.1012271 (PMC13492815; doi:10.1371/journal.pgen.1012271)
Supplement: Supplementary file 10 [file pgen.1012271.s010.docx]

**S10 Fig. Observed and expected heterozygosity are significantly associated with GERP score, following a linear trend.** General additive model of **A)** observed heterozygosity (H_O_) in the MASPOT panel and **B)** expected heterozygosity (H_S_) in the MASPOT panel as a function of GERP score. The second axes show the effects of GERP score on H_O_ and H_S_, respectively.
