# Supplementary material for: Genomic constraint and hypervariability correlate with fitness effects in elite tetraploid potato breeding material
Source: PLoS Genet. 2026 Aug 11;22(8):e1012271. doi: 10.1371/journal.pgen.1012271 (PMC13492815; doi:10.1371/journal.pgen.1012271)
Supplement: Supplementary file 11 [file pgen.1012271.s011.docx]

**S11 Fig. Observed heterozygosity was consistently lower than expected heterozygosity across all marker types.** Mean observed heterozygosity (H_O_) [top] and gene diversity (expected heterozygosity [H_S_]) [bottom] across all grouped by marker annotation. The mean heterozygosity is shown as a black dot.
