# Supplementary material for: Genomic constraint and hypervariability correlate with fitness effects in elite tetraploid potato breeding material
Source: PLoS Genet. 2026 Aug 11;22(8):e1012271. doi: 10.1371/journal.pgen.1012271 (PMC13492815; doi:10.1371/journal.pgen.1012271)
Supplement: Supplementary file 12 [file pgen.1012271.s012.docx]

**S12 Fig. Observed heterozygosity is significantly associated with GERP score, following a linear trend in the landrace panel.** General additive model of observed heterozygosity (H_O_) in the landrace panel of Wu et al., (2023) as a function of GERP score. The observed heterozygosity was computed for 6,850,272 SNPs with multiple sequence alignment depth > 50 and neutral score > 2. The second axis show the effects of GERP score on H_O_.
