# Supplementary material for: Genomic constraint and hypervariability correlate with fitness effects in elite tetraploid potato breeding material
Source: PLoS Genet. 2026 Aug 11;22(8):e1012271. doi: 10.1371/journal.pgen.1012271 (PMC13492815; doi:10.1371/journal.pgen.1012271)
Supplement: Supplementary file 13 [file pgen.1012271.s013.docx]

**S13 Fig. The inbreeding coefficient across had high variability across marker types.** Mean observed inbreeding coefficient (F_IS_) across all markers (left), deleterious markers (center), and hypervariable markers (right) grouped by marker annotation. The mean inbreeding coefficient is shown as a black dot.
