# Supplementary material for: Genomic constraint and hypervariability correlate with fitness effects in elite tetraploid potato breeding material
Source: PLoS Genet. 2026 Aug 11;22(8):e1012271. doi: 10.1371/journal.pgen.1012271 (PMC13492815; doi:10.1371/journal.pgen.1012271)
Supplement: Supplementary file 14 [file pgen.1012271.s014.docx]

**S14 Fig. Complementation of deleterious variants compared to non-deleterious mutations matched that compared to other mutations.** Comparing the percentage of heterozygous genotypes (simplex-triplex) of non-deleterious mutations [GERP < 2] with that of mildly [2 ≤ GERP < 2.75], moderately [2.75 ≤ GERP < 3.5], and highly [GERP ≥ 3.5] deleterious mutations in the MASPOT panel. The dashed gray line indicates “*y = x*”. The solid lines represent linear regressions within groups of points and are shown with 95 % confidence intervals in gray. Within each color group each point is one MASPOT F_1_ clone. (mild: t = 147.95, *p*-value < 2.2*10^-16^; moderate: t = 148.99, *p*-value < 2.2*10^-16^; high: t = 131.45, *p*-value < 2.2*10^-16^; paired Student’s t-test).
