# Supplementary material for: Genomic constraint and hypervariability correlate with fitness effects in elite tetraploid potato breeding material
Source: PLoS Genet. 2026 Aug 11;22(8):e1012271. doi: 10.1371/journal.pgen.1012271 (PMC13492815; doi:10.1371/journal.pgen.1012271)
Supplement: Supplementary file 15 [file pgen.1012271.s015.docx]

**S15 Fig. The patterns of complementation of alleles at constrained and hypervariable loci were consistent for high-read depth genotypes alone.** **A)** Comparing the percentage of heterozygous genotypes (simplex-triplex) of other mutations [-4 < GERP < 2] with that of mildly [2 ≤ GERP < 2.75], moderately [2.75 ≤ GERP < 3.5], and highly [GERP ≥ 3.5] deleterious mutations in the MASPOT panel for markers filtered for read depth > 20. (mild: t = 84.89, *p*-value < 2.2*10^-16^; moderate: t = 89.07, *p*-value < 2.2*10^-16^; high: t = 93.87, *p*-value < 2.2*10^-16^; paired Student’s t-test). **B)** The same comparison for mildly [-5.5 < GERP ≤ -4], moderately [-7 < GERP ≤ -5.5], and highly [GERP ≤ -7] hypervariable variants. (mild: t = -114.35, *p*-value < 2.2*10^-16^; moderate: t = -103.66, *p*-value < 2.2*10^-16^; high: t = -101.98, *p*-value < 2.2*10^-16^; paired Student’s t-test) The dashed gray line indicates “*y = x*”. The solid lines represent linear regressions within groups of points and are shown with 95 % confidence intervals in gray. Within each color group each point is one MASPOT F_1_ clone.
