# Supplementary material for: Genomic constraint and hypervariability correlate with fitness effects in elite tetraploid potato breeding material
Source: PLoS Genet. 2026 Aug 11;22(8):e1012271. doi: 10.1371/journal.pgen.1012271 (PMC13492815; doi:10.1371/journal.pgen.1012271)
Supplement: Supplementary file 16 [file pgen.1012271.s016.docx]

**S16 Fig. The ratio of homozygosity depreciates with GERP score at constrained loci and increases with GERP score at hypervariable loci. A)** Comparing the percentage of homozygous genotypes and heterozygous genotypes (simplex-triplex) across mildly [2 ≤ GERP < 2.75], moderately [2.75 ≤ GERP < 3.5], and highly [GERP ≥ 3.5] deleterious mutations in the MASPOT panel. (mild: t = 301.3, *p*-value < 2.2*10^-16^; moderate: t = 310.38, *p*-value < 2.2*10^-16^; high: t = 276.17, *p*-value < 2.2*10^-16^, paired Student’s t-test). **B)** The same comparison for mildly [-5.5 < GERP ≤ -4], moderately [-7 < GERP ≤ -5.5], and highly [GERP ≤ -7] hypervariable variants. (mild: t = -345.53, *p*-value < 2.2*10^-16^; moderate: t = -315.58, *p*-value < 2.2*10^-16^; high: t = -303.33, *p*-value < 2.2*10^-16^, paired Student’s t-test). The solid lines represent linear regressions within groups of points and are shown with 95 % confidence intervals in gray. Within each color group each point is one MASPOT F_1_ clone.
