# Supplementary material for: Genomic constraint and hypervariability correlate with fitness effects in elite tetraploid potato breeding material
Source: PLoS Genet. 2026 Aug 11;22(8):e1012271. doi: 10.1371/journal.pgen.1012271 (PMC13492815; doi:10.1371/journal.pgen.1012271)
Supplement: Supplementary file 17 [file pgen.1012271.s017.docx]

**S17 Fig. Partial correlations between heterozygous deleterious load and phenotypes.** Partial correlations between clone phenotype and the per-individual residual heterozygous deleterious load (after fitting the homozygous load and correcting for population structure [fitting principal components 1-3 of the genomic relationship matrix]) among the MASPOT panel clones for each trait. Pearson partial correlation coefficients and Pearson partial correlation test *p*-values are shown. The solid black line indicates the linear regression between phenotype and residual load. Gray shadows indicate 95 % confidence intervals.
