# Supplementary material for: Genomic constraint and hypervariability correlate with fitness effects in elite tetraploid potato breeding material
Source: PLoS Genet. 2026 Aug 11;22(8):e1012271. doi: 10.1371/journal.pgen.1012271 (PMC13492815; doi:10.1371/journal.pgen.1012271)
Supplement: Supplementary file 25 [file pgen.1012271.s025.docx]

**S25 Fig. Manhattan plots of GWAS of length.** Manhattan plots of -log_10_(*p*-value) GWAS results of 6 models for tuber length. From top to bottom: the baseline model, the total alternative allele model, the heterozygous deleterious load model, the homozygous deleterious load model, the heterozygous hypervariable load model, and the homozygous hypervariable load model. The significance threshold with Bonferroni correction is indicated with a horizontal line.
