# Supplementary material for: Genomic constraint and hypervariability correlate with fitness effects in elite tetraploid potato breeding material
Source: PLoS Genet. 2026 Aug 11;22(8):e1012271. doi: 10.1371/journal.pgen.1012271 (PMC13492815; doi:10.1371/journal.pgen.1012271)
Supplement: Supplementary file 35 [file pgen.1012271.s035.docx]

**S35 Fig. QQ plot comparison of GWAS models for size.** QQ plots of -log_10_(*p*-value) GWAS results of the baseline model against [Expected *p*] (from left to right) -log_10_(*p*-value) GWAS results of the total alternative allele model (fitting the number of derived alleles), the homozygous deleterious load model (fitting the homozygous deleterious load), the heterozygous deleterious load model (fitting the heterozygous deleterious load), the homozygous hypervariable load model (fitting the homozygous hypervariable load),or the heterozygous hypervariable load model (fitting the heterozygous hypervariable load) [Observed *p*] for the trait tuber size. The black line indicates $x=y$.
