# Supplementary material for: Genomic constraint and hypervariability correlate with fitness effects in elite tetraploid potato breeding material
Source: PLoS Genet. 2026 Aug 11;22(8):e1012271. doi: 10.1371/journal.pgen.1012271 (PMC13492815; doi:10.1371/journal.pgen.1012271)
Supplement: Supplementary file 36 [file pgen.1012271.s036.docx]

**S36 Fig. SNP density of the MASPOT panel follows genome-wide gene density.** Heatmap of marker density in 1 Mb windows for each of the 12 chromosomes. The color gradient denotes window marker count.
