# Supplementary material for: Genomic constraint and hypervariability correlate with fitness effects in elite tetraploid potato breeding material
Source: PLoS Genet. 2026 Aug 11;22(8):e1012271. doi: 10.1371/journal.pgen.1012271 (PMC13492815; doi:10.1371/journal.pgen.1012271)
Supplement: Supplementary file 37 [file pgen.1012271.s037.docx]

**S37 Fig. Principal component analysis of the genomic relationship matrix showed some family structure.** Principal component analysis of the genomic relationship matrix colored by mother (left) and father (right). Principal component 1 is plotted against principal component 2 in the top and against principal component 3 in the bottom. Principal components 1-3 explain 20.3 %, 11.06 %, and 9.99 % of the total explained variance, respectively.
