# Supplementary material for: Genomic constraint and hypervariability correlate with fitness effects in elite tetraploid potato breeding material
Source: PLoS Genet. 2026 Aug 11;22(8):e1012271. doi: 10.1371/journal.pgen.1012271 (PMC13492815; doi:10.1371/journal.pgen.1012271)
Supplement: Supplementary file 39 [file pgen.1012271.s039.docx]

**S1 Table. Genomic prediction covariate coefficients for dry matter content.**

| **Covariate** | **Coefficient** |
| --- | --- |
| **Total alt. alleles** | 1.3793353 |
| **Deleterious homozygous load** | 1.534686e-03 |
| **Deleterious heterozygous load** | 0.4779085 |
| **Hypervariable homozygous load** | 0.8653137 |
| **Hypervariable heterozygous load** | 0.4586444 |

Estimated coefficients of covariates fitted in genomic prediction models of dry matter content (for models with that covariate alone) with significance indicated based on Type III Wald test *p*-values using a 0.05 significance level (*).
