# Supplementary material for: Genomic constraint and hypervariability correlate with fitness effects in elite tetraploid potato breeding material
Source: PLoS Genet. 2026 Aug 11;22(8):e1012271. doi: 10.1371/journal.pgen.1012271 (PMC13492815; doi:10.1371/journal.pgen.1012271)
Supplement: Supplementary file 40 [file pgen.1012271.s040.docx]

**S2 Table. Genomic prediction covariate coefficients for yield.**

| **Covariate** | **Coefficient** |
| --- | --- |
| **Total alt. alleles** | 0.3303058 |
| **Deleterious homozygous load** | 6.650755 (*) |
| **Deleterious heterozygous load** | 2.542567 |
| **Hypervariable homozygous load** | 8.355769 (*) |
| **Hypervariable heterozygous load** | 8.517871 (*) |

Estimated coefficients of covariates fitted in genomic prediction models of yield (for models with that covariate alone) with significance indicated based on Type III Wald test *p*-values using a 0.05 significance level (*).
