# Supplementary material for: Genomic constraint and hypervariability correlate with fitness effects in elite tetraploid potato breeding material
Source: PLoS Genet. 2026 Aug 11;22(8):e1012271. doi: 10.1371/journal.pgen.1012271 (PMC13492815; doi:10.1371/journal.pgen.1012271)
Supplement: Supplementary file 43 [file pgen.1012271.s043.docx]

**S5 Table. Genomic prediction covariate coefficients for tubers/plant.**

| **Covariate** | **Coefficient** |
| --- | --- |
| **Total alt. alleles** | 1.266785 |
| **Deleterious homozygous load** | 1.685905 |
| **Deleterious heterozygous load** | 0.1053026 |
| **Hypervariable homozygous load** | 1.135115 |
| **Hypervariable heterozygous load** | 1.683699 |

Estimated coefficients of covariates fitted in genomic prediction models of tubers/plant (for models with that covariate alone) with significance indicated based on Type III Wald test *p*-values using a 0.05 significance level (*).
