# Supplementary material for: Genomic constraint and hypervariability correlate with fitness effects in elite tetraploid potato breeding material
Source: PLoS Genet. 2026 Aug 11;22(8):e1012271. doi: 10.1371/journal.pgen.1012271 (PMC13492815; doi:10.1371/journal.pgen.1012271)
Supplement: Supplementary file 47 [file pgen.1012271.s047.docx]

**S9 Table. Imputation accuracies with 3 % and 7 % introduced missingness.**

|  | **3 % masked** | | **7 % masked** | |
| --- | --- | --- | --- | --- |
| **Chromosome** | **Correlation** | **% true masked** | **Correlation** | **% true masked** |
| 1 | 0.878 | 2.865 | 0.877 | 6.675 |
| 2 | 0.883 | 2.872 | 0.882 | 6.685 |
| 3 | 0.875 | 2.873 | 0.874 | 6.688 |
| 4 | 0.885 | 2.86 | 0.884 | 6.656 |
| 5 | 0.885 | 2.858 | 0.883 | 6.653 |
| 6 | 0.882 | 2.865 | 0.881 | 6.67 |
| 7 | 0.889 | 2.862 | 0.888 | 6.664 |
| 8 | 0.884 | 2.858 | 0.883 | 6.654 |
| 9 | 0.886 | 2.868 | 0.884 | 6.68 |
| 10 | 0.89 | 2.865 | 0.889 | 6.671 |
| 11 | 0.883 | 2.873 | 0.881 | 6.69 |
| 12 | 0.884 | 2.857 | 0.882 | 6.651 |

Mean Pearson correlation for five repeats of imputation with 3 % and 7 % masked genotype entries, respectively. The mean true missing rate, i.e. the applied masking subtracted the masked entries with missing data, is shown on the right of the Pearson correlations.
