# Supplementary material for: Genomic constraint and hypervariability correlate with fitness effects in elite tetraploid potato breeding material
Source: PLoS Genet. 2026 Aug 11;22(8):e1012271. doi: 10.1371/journal.pgen.1012271 (PMC13492815; doi:10.1371/journal.pgen.1012271)
Supplement: Supplementary file 48 [file pgen.1012271.s048.docx]

**Table S10. Genomic inflation factors for GWAS of each trait.**

|  | **Dry matter content** | **Yield** | **Senescence** | **Flesh color** | **Tubers/plant** | **Length** | **Diameter** | **Size** |
| --- | --- | --- | --- | --- | --- | --- | --- | --- |
| $\boldsymbol{\lambda}_{\boldsymbol{gc}}$ | 1.14 | 1.10 | 1.04 | 1.06 | 1.09 | 1.11 | 1.16 | 1.12 |

Genomic inflation factors ($\lambda_{gc}$) for the baseline GWAS model for each of the analyzed traits.
