# Supplementary material for: Genomic constraint and hypervariability correlate with fitness effects in elite tetraploid potato breeding material
Source: PLoS Genet. 2026 Aug 11;22(8):e1012271. doi: 10.1371/journal.pgen.1012271 (PMC13492815; doi:10.1371/journal.pgen.1012271)
Supplement: Supplementary file 49 [file pgen.1012271.s003.docx]

**S3 Fig. GERP score density.** Density plot of GERP scores of the final 97,815 SNP set.
